# Assessment of Swallowing Disorders, Nutritional and Hydration Status, and Oral Hygiene in Students with Severe Neurological Disabilities Including Cerebral Palsy

**DOI:** 10.3390/nu13072413

**Published:** 2021-07-14

**Authors:** Alicia Costa, Alberto Martin, Viridiana Arreola, Stephanie A. Riera, Ana Pizarro, Cristina Carol, Laia Serras, Pere Clavé

**Affiliations:** 1 Unitat d’Exploracions Funcionals Digestives CSdM-UAB, Hospital de Mataró, 08304 Mataró, Spain; amartinma@csdm.cat (A.M.); oarreola@csdm.cat (V.A.); eriera@csdm.cat (S.A.R.); 2 Unitat de Dietètica i Nutrició, Hospital de Mataró, 08304 Mataró, Spain; 3 Departament de Medicina, Universitat Autònoma de Barcelona, 08035 Cerdanyola del Vallès, Spain; 4 Centro de Investigación Biomédica en Red de Enfermedades Hepáticas y Digestivas (CIBERehd), Instituto de Salut Carlos III, 28029 Madrid, Spain; 5 Departament de Periodòncia, Facultat d’Odontologia de Barcelona UIC, 08195 Sant Cugat del Vallès, Spain; od079353@uic.es; 6 Escola l’Arboç, Fundació El Maresme, 08301 Mataró, Spain; ccarol@fundaciomaresme.cat; 7 Unitat de Suport Tècnic, Fundació El Maresme, 08301 Mataró, Spain; lserras@fundaciomaresme.cat; 8 Fundació de Recerca en Gastroenterologia (Furega), 08008 Barcelona, Spain

**Keywords:** neurological disability, cerebral palsy, dysphagia, malnutrition, dehydration, dietary intakes, swallowing disorders, feeding difficulties, oral health

## Abstract

Background: Special needs schools (SNS) educate children and young people with major neurological disabilities who are at high risk of oropharyngeal dysphagia (OD) and malnutrition (MN). We aimed to assess the prevalence of OD, MN, dehydration (DH), and oral health (OH) in students at an SNS. Methods: A cross-sectional observational study was conducted at SNS L’Arboç, Catalonia, Spain. We assessed (a) demographics, health status, comorbidities, and gross motor function classification system (GMFCS), (b) swallowing function, oral-motor evaluation, masticatory capacity, and EDACS classification for eating and drinking abilities, (c) nutritional and DH status (anthropometry, bioimpedance and dietary records), and (d) OH (Oral Hygiene Index Simplified). Results: A total of 33 students (mean age 13.3 years; 39.4% level V of GMFCS) were included. Main diagnosis was cerebral palsy at 57.6%. All students presented OD, 90.6% had impaired safety, 68.7% were at levels II–III of EDACS, and 31.3% required PEG; furthermore, 89.3% had chronic MN, 21.4% had acute MN, 70% presented intracellular DH, and 83.9% presented impaired OH. Conclusion: MN, DH, OD, and poor OH are highly prevalent conditions in students with cerebral palsy and other neurological disabilities and must be specifically managed through nutritional and educational strategies. The multidisciplinary team at SNS should include healthcare professionals specifically trained in these conditions.

## 1. Introduction

Special needs schools (SNS) for children and young people with major neurological disabilities (ND) and cerebral palsy (CP) have as a main objective the promotion to the highest degree of students’ personal autonomy and social integration by developing their physical, affective, cognitive, communicative, moral, civic, and social insertion abilities. Multidisciplinary teams at SNS in Catalonia (Spain) are composed of educational staff including special education teachers, social educators, SLP teachers and physiotherapists. L’Arboç SNS also has a social worker, a nurse, a neurologist, and a psychologist. SNS educate children with major associated health problems (musculoskeletal disorders, intellectual disability, epilepsy, swallowing disorders, and malnutrition (MN)). In early childhood and adolescence, these populations are more likely to suffer from poor nutrition than during adulthood because of the high nutritional demands needed for growth and development. Providing adequate nutrition and hydration to these students is essential to promote their maximal level of both physical and cognitive development [1]. Oropharyngeal dysphagia (OD) is an extremely common digestive disorder amongst children with ND and CP, with a reported prevalence in excess of 90% [2,3]. The main complications of OD include respiratory infections, aspiration pneumonia, MN, and dehydration (DH) [4,5]. Respiratory diseases are the leading cause of death in patients with severe intellectual disability and severe developmental disability [6,7,8].

Feeding and swallowing difficulties in children with ND and CP severely affect nutrient intake, which leads to MN, DH, alterations in linear growth, and micronutrient deficiency [2,3,4,5]. Poor nutritional status may detrimentally impact health and physical and cognitive development [5,6,7,8]. CP describes a group of permanent disorders of movement and posture development attributed to nonprogressive disturbances that occurred in the developing fetal or infant brain [3]. CP can be caused by injury to the brain at birth, during the early stages of development in the womb, or during the first two years of life. Head injuries, infections such as meningitis, and other forms of brain damage occurring in the first months or years of life are the main causes of acquired CP [1]. Up to four types of CP have been described, determined by timing, site, type, and extent of the brain lesion: (a) spastic, the most common form (70–80%) affecting motor cortex, (b) dyskinetic arising from the basal ganglia and affecting less than 10% of cases, (c) ataxic arising from cerebellum damage, also affecting less than 10% of cases, and (d) mixed types with a combination of damage [1]. The prevalence of CP in Europe is 2.08 per 1000 live births [9], and CP is considered a relatively rare disorder there [1]. Primary neurological impairments might influence not only physical and mental capacities but also neural pathways leading to dysphagia, vomiting, swallowing deficits, gastroesophageal reflux, aspiration, and constipation [10,11,12,13,14].

Motor disorders in CP are often accompanied by alterations in sensation, cognition, and communication, as well as by secondary musculoskeletal problems [15], and they are classified by the GMFCS into five subgroups according to their severity [16]. The severity of GMFCS also correlates with the severity of swallowing difficulties [17,18]. The most severe complication of swallowing disorders is respiratory infection, which occurs when food, liquid, or oropharyngeal secretions, together with microorganisms, are aspirated into the respiratory tract [19,20]. OH is closely related to OD because the oropharynx is a constant source and reservoir of microorganisms responsible for respiratory infections in patients with OD [19,20]. In general, young people and children with ND present greater oral pathology with poorer OH and gingival health and a higher incidence of dental caries than children without ND [21]. The severity of neurological impairment correlates with increased risk of developing dental disease [22]. For these reasons, it is recommended that children with ND are assessed for OD to avoid safety impairments, and they should receive early and regular dental care, in order to minimize the reservoir of oral pathogens [21].

Regarding MN, studies have shown high prevalence among children and young people with ND compared to the general population [23,24]. MN has been observed in 46% and 90% of children with CP [25,26] and, in the most severe types, the prevalence increases due to the severity of cognitive and motor impairments, OD, and alterations in orolingual facial motricity leading to eating difficulties [13,14,26,27,28,29]. Higher rates of MN are reported among children with bilateral spasticity, dyskinesia, and spastic quadriplegia [23,26,30,31]. Poor nutritional status is associated with growth impairment, increased spasticity, and irritability [14,30], in addition to further risk of adverse cognitive and health outcomes including respiratory complications and mortality. The etiology of MN in children with neurological impairment is multifactorial, and one of the major contributors is OD and subsequent insufficient caloric and nutrient intake [32,33]. When nutritional intake is insufficient to cover nutritional requirements or the oral route is not safe, children need enteral nutrition through percutaneous endoscopic gastrostomy (PEG) to feed. The prevalence of children with CP with a gastrostomy tube is 11% in European countries [34].

Nutritional assessment and support are an essential part of the care of people with ND and CP who have extremely complex and challenging needs [24]. Adequate nutritional support may restore linear growth, normalize weight, decrease irritability and spasticity, improve wound healing, reduce the frequency of hospitalization, and increase social participation, thus improving overall health and quality of life [35]. Early screening and management through nutritional and compensatory strategies of OD and MN in SNS can be an effective strategy to improve physical and intellectual development, clinical outcomes, and quality of life for their students [36,37,38].

L’Arboç is an SNS for children and young people with multiple and major ND, located in the city of Mataró, Catalonia, Spain. The school takes care of 50 students, from 3 to 23 years of age, with CP, severe ND, and psychomotor disabilities that interfere with their development and learning processes [39]. Awareness and management of OD, MN, and OH in SNS in Spain is scarce and must be improved. As most students have severe neurological impairments, we hypothesized that most of them would have dysfunctional swallowing. We believe the introduction of a program mainly based on the management of OD and MN in children attending these schools and the education of their caregivers in the same will improve students’ hydration and nutritional status, reduce complications associated with OD (respiratory infections and hospital readmissions), and improve the knowledge of parents, caregivers, and professionals at these schools on the management of these relevant conditions.

The aim of this study was to assess the prevalence of swallowing and feeding disorders and oral health impairments in students at L’Arboç SNS and their relation to students’ nutritional and hydration status. This is the first step of a program that includes (a) a transversal descriptive study that will be repeated at the end of each school year, assessing OD, nutritional status, and oral health, (b) the implementation of a nutritional intervention including adaptations in texture, caloric and protein content, and palatability during meals at school and at home, (c) a hydration program including regular services, fluid-thickening agents, and special cups at school and home, and (d) an educational program on the clinical relevance and management of OD and MN in these children for parents, caregivers, and all professionals at L’Arboç school (Supplementary Material S1).

## 2. Patients and Methods

### 2.1. Study Design

A cross-sectional observational study was conducted between 1 February 2019 and 30 May 2019 in an SNS, L’Arboç School, (Mataró, Catalonia, Spain). Assessments and classifications included (a) demographics, health status, comorbidities, medication, and GMFCS, (b) swallowing function (with the Volume Viscosity Swallowing Test (V-VST)), and oral-motor evaluation, masticatory capacity, and EDACS for eating and drinking ability, (c) OH status according to the Oral Hygiene Index Simplified (OHIS), and (d) nutritional and hydration status (anthropometry, bioimpedance (BIA) and dietary records). The multidisciplinary team for these assessments included a dietitian, a speech and language therapist, a nurse, and a dentist. 

### 2.2. Inclusion Criteria

Main inclusion criteria were to be students of L’Arboç School (Mataró, Catalonia, Spain) and to have informed consent of the parents or legal guardians. Exclusion criteria were children whose parents or caregivers did not provide informed consent. The study protocol was approved by the ethics review board of the Hospital de Mataró (code 01/19). 

### 2.3. Demographics, Health Status, Comorbidities, Medication, and GMFCS

Data on demographics (age, gender, reference health center, educational level, and social class of the parents or main caregiver), clinical factors (neurological diagnosis, comorbidities, GMFCS level and associated impairments, and hospital admissions during last 12 months), and chronic pharmacologic treatments were collected from the electronic medical records (HC3). The GMFCS is a five-level classification that differentiates children with CP on the basis of the child’s current gross motor abilities, limitations in gross motor function, and need for assistive technology and wheeled mobility [40]. The GMFCS applies to all types of CP and all levels of severity, and it takes into account the age of the person; a higher level in GMFCS denotes more severe CP [41]. In our study, GMFCS was performed by a neurologist.

### 2.4. Oral-Motor Evaluation, Masticatory Capacity, and Swallowing Function

The SLP assessed (a) oral-motor assessment and meal observation, (b) prevalence and severity of OD by clinical swallowing evaluation using the V-VST, (c) severity of their feeding impairment following the EDACS, and (d) masticatory capacity.

#### 2.4.1. Oral-Motor Evaluation and Meal Observation

Prior to the evaluation of the oral-motor capacity, the parents or the person responsible for feeding the students were interviewed about (a) the length of time it takes the students to eat, (b) the posture of students when meals are administered, and (c) the students’ degree of autonomy.

In children who were reported to be able to eat some food in pieces, a direct observation was made for (a) food aversion, (b) impaired lip seal, (c) tongue protrusion, (d) accumulation of the bolus in the mouth, (e) uncontrolled bolus spillage into the pharynx with three viscosities (low, medium, and high), (f) hyperreactivity at mealtimes, (g) feeding rhythm (slow, medium and quick), (h) eating autonomy, and (i) cervical hyperextension during the meal. 

The difficulties with sucking, swallowing, chewing, drooling, independent feeding, and feeding problems were graded as “present” or “absent”. Tongue, jaw and mouth function, swallowing function [42], swallowing assessment [43,44], aspiration, and choking were also evaluated during the intakes. 

Food texture provided to each student by their families and teachers was classified into four groups: Texture B (thin purée, traditional food processed, diluted) through PEG, Texture C (thick purée), Texture E (fork-mashable) from the National Descriptors for Texture Modification by the British Dietetic Association [45], and normal diet (diets without any textural modification). Data of those students who were fed with specific products for oral enteral nutrition were also collected. Following the evaluation, textures consumed by the students were compared with the textures recommended for each of the students to check their suitability.

#### 2.4.2. Clinical Swallow Evaluation for OD (V-VST)

The V-VST is a validated clinical assessment tool for OD that uses three volumes (5, 10, and 20 mL) and viscosities (middle (250 mPa·s), low (liquid), and high (800 mPa·s)). In this case, taking into account the age, the high comorbidity of the students, and the risk of premature bolus spillage into the pharynx, it was decided to adapt the V-VST by applying 5, 10, and 15 mL. It uses a pulse-oximeter to detect silent aspirations, to evaluate clinical signs of impaired efficacy and safety of swallow [46,47]. Diagnostic sensitivity and specificity for OD are 90% and 80%, respectively, and the reliability of V-VST is also high with an overall Kappa value of 0.77 (95% CI 0.65–0.89) [47]. For the V-VST evaluation, Nutilis Clear thickener was used (Nutilis Clear, Nutricia N.V., Zoetermeer, the Netherlands). It is a xanthan-gum-based thickener mixed with maltodextrin and guar gum. Viscosity levels (250 mPa·s and 800 mPa·s) were chosen in accordance with a clinical trial performed by our group [48]. For those students who were exclusively fed and hydrated by PEG, the last videofluoroscopy (VFS) was taken into account to determine their swallowing status. 

#### 2.4.3. Eating and Drinking Ability Classification System (EDACS)

The EDACS describes the functional eating and drinking abilities of children with CP aged 3 years and older, using five distinct levels. It refers to key features of ‘safety’ (aspiration and choking) and ‘efficiency’ (amount of food lost and time taken to eat) [16,49]. The EDACS provides a valid and reliable system for classifying eating and drinking performance of people with CP, for use in both clinical and research contexts, classified according to the following levels: Level I, eats and drinks safely and efficiently; Level II, eats and drinks safely but with some limitations to efficiency; Level III, eats and drinks with some limitations to safety, maybe limitations to efficiency; Level IV, eats and drinks with significant limitations to safety; Level V, unable to eat or drink safely, whereby tube feeding may be considered to provide nutrition.

EDACS was developed in four stages in consultation with individuals with CP, parents, and health professionals: Stage1, drafting informed by the literature and clinical experience; Stage 2, modification by nominal groups; Stage 3, refinement in an international Delphi survey; Stage 4, testing of agreement and reliability between classifications made by SLPs, as well as between SLPs and parents [16,49].

#### 2.4.4. Masticatory Capacity

Some items evaluated during meal observation (tongue protrusion, masticatory and tongue movements, and mouth pocketing and duration) and some questions of the caregivers interview (duration and fatigue) were used to help define the masticatory capacity of our participants. To complement it, in cases where students were eating a regular diet or type E, a more exhaustive assessment was conducted in order to evaluate the capacity of the participants to chew and grind food prior to swallowing. They were given one-quarter of a Marie-type biscuit to observe their chewing efficiency, taking into account if there was visible activity of the tongue and rotational mobility of the jaw, if there were bilateral and alternate chewing cycles, and if they made lingual movements before swallowing. Lastly, it was also observed if several swallows were needed for the small proportion of the biscuit given and if oral residue remained, as well as the total time taken to eat the piece of biscuit.

### 2.5. Oral Health (OH) Assessment 

Oral examinations were performed by a dentist and included occlusion assessment, oral hygiene, and periodontal assessment, as described in our previous studies on adults [50,51].

#### 2.5.1. Occlusion Assessment

Facial and malocclusion analysis were analyzed by Angle’s classification of malocclusion [52]. This classification is based on the relationship between the mesiobuccal cusp of the maxillary first permanent molar and the buccal groove of the mandibular first permanent molar. According to this classification, three types of malocclusion were obtained.

#### 2.5.2. Oral Hygiene

Oral hygiene examination was evaluated according to the simplified Oral Hygiene Index (OHI-s). It is composed of the sum of two indices, Debris Index (DI-S) and Calculus Index (CI-S). Six indicator teeth are examined for soft deposits and calculus. DI-S and CI-S values range from 0–3 depending on the coverage of each tooth. The final value of OHI-s is obtained by adding DI-S and CI-S, for a score between 0 and 6 [53].

#### 2.5.3. Periodontal Assessment

Periodontal assessment was evaluated according to the new classification of periodontal and peri-implant conditions [54]. Stage I to IV of periodontitis is defined on the basis of severity (primarily periodontal breakdown with reference to root length and periodontitis-associated tooth loss), complexity of management (pocket depth, infrabony defects, furcation involvement, tooth hypermobility, masticatory dysfunction), and extent (localized or generalized). Grade of periodontitis is estimated with direct or indirect evidence of progression rate in three categories: slow, moderate, and rapid progression (Grade A–C) [55].

### 2.6. Nutritional and Hydration Assessment 

The assessment of nutritional and hydration status included anthropometric measurements, BIA studies, and dietary intake registries, which were assessed by nutritionists.

#### 2.6.1. Anthropometric Measurements

(a) Body weight and height: The students’ weight was measured using a wheelchair scale (Detecto, Model 6550 Fold-up Portable Wheelchair Scale, Webb City, MO, USA, 2010), the weight of which was later subtracted. Other children were weighed together with a parent and then the parent’s weight was subtracted. Following ESPGHAN guidelines, height was estimated from the knee height by using the Almond equation [56] for children younger than 12 years (*n* = 15) and the Chumlea equation [57,58] for students over 12 years of age. The knee height was measured with the child seated or in a supine position, with the knee and ankle at a 90° angle. Body mass index (BMI) was calculated from the weight and height of the student (BMI = weight (kg)/height (m^2^)).

(b) Skinfold thickness: Subscapular skinfold thickness, triceps skinfold thickness, and abdomen skinfold thickness were measured using a skinfold caliper (0.2 mm precision) (Holtain^®^ skinfold caliper, London, UK), averaging the three measurements. Triceps and subscapular skinfolds and bioimpedance (BIA) have been shown to be accurate and noninvasive methods to estimate body fat percentage in children with CP [59]. Slaughter equations based on the triceps and subscapular skinfolds were used to estimate body fat percentage for each individual [60]. Cutoffs for body fat (%) were based on the National Health and Nutrition Examination Survey (NHANES) [61].

#### 2.6.2. Bioimpedance 

Body composition and hydration status were assessed with a body composition analyzer (InBody S10^®^; Biospace Co., Ltd., Seoul, Korea). This device is based on multifrequency bioelectrical impedance analysis, BIA (1 kHz, 5 kHz, 50 kHz, 250 kHz, 500 kHz, and 1000 kHz) and contains eight-point tactile electrodes that were attached to the left and right thumb, middle finger, and ankles [62]. All children were explored 2 h after they finished their breakfast. The majority of children were evaluated while sitting in their own wheelchair, whereas some were lying on a bed. 

Main compartments analyzed were (a) skeletal muscle mass, body fat mass, and fat-free mass, and (b) body water (intracellular; extracellular; total; body water ratio, ECW/TBW), body cell mass, and phase angle (PhA). Reference values of mean PhA in healthy children were obtained from the Nogueira and Bosy-Westphal studies [63,64].

The normal range of the overall population was obtained from the mean of the normality intervals according to age provided by the BIA for each student [62].

#### 2.6.3. Nutritional and Food Intake Assessment. Dietary Records

Food intake was registered with 24 h recall and 7 day feeding history and used to quantify the calorie and nutrient intake. Dietary habits were assessed by means of a food frequency questionnaire. The results were compared with the recommendations for a student’s balanced diet according to the Mediterranean Diet [65].

### 2.7. Data Analysis of Nutritional Variables

To assess the nutritional status and to identify those students with MN, we used (a) growth charts based on *z*-score (WHO) [66], (b) growth charts based on percentiles [27], and (c) Waterlow classification [67].

#### 2.7.1. Grow Charts Based on *z*-Score (WHO) for Healthy Children

This analysis indicates the ideal growth based on a standardized reference population (WHO growth standards) [66]. *z*-Scores from three standard indices were used to assess nutritional status: (a) weight for age *z*-score (WAZ) to measure overall nutritional status, (b) height for age *z*-score (HAZ) to measure chronic MN, and (c) BMI for age (BAZ) to measure acute MN. The *z*-score for weight was calculated using WHO Anthro (version 3.2). WHO Anthro analyzes children up to 10 years old, as the weight of children above this age group is influenced by their height [66]. The *z*-scores for height and BMI were calculated using WHO AnthroPlus software, to monitor the growth of school-age children and adolescents from 5 to 19 years old. The calculated *z*-scores for each of the indices were classified into three categories according to WHO cutoff points to measure the severity of MN among students: (a) overnutrition (>+3 SD); (b) normal (+2 SD to −2 SD); (c) moderate undernutrition (<−2 SD to −3 SD); (d) severe undernutrition (<−3 SD) [14,33].

#### 2.7.2. Growth Charts Based on Percentiles (Brooks) for Children with CP

The anthropometric profile was also calculated through the specific growth curves for children with CP based on GMFCS according to Brooks references. Weight/age, height/age, and BMI expressed in percentiles were calculated from anthropometric measurements. Low weight was defined as BMI ≤p10 and nutritional risk if weight/age was less than p5 (in children with GMFCS I and II) or less than p20 (in children with GMFCS III to V). These growth charts proposed cutoff points of the weight/age index associated with higher morbidity and mortality [27].

#### 2.7.3. Waterlow Classification

The Waterlow Index is a system for classifying protein-energy MN in children. It is a widely used index in clinical practice for children with neurological impairment. It allows for the distinction between “wasting” (reflects acute MN and is based upon weight-for-height) and “stunting” (which reflects chronic MN and is based upon height-for-age). Cases with MN were further categorized in terms of severity on the basis of percentage weight-for-height (normal (≥90%), mild (80–89%), moderate (70–79%), severe (<70%)) and percentage height-for-age (normal (≥95%), mild (90–94%), moderate (85–89%), severe (<85%)) parameters [14,33]. The anthropometric red-flag warning signs for MN recommended by the European Society for Pediatric Gastroenterology, Hepatology, and Nutrition (ESPGHAN) work group for children with neurological impairment were also considered [32].

#### 2.7.4. Assessment of Energy Expenditure

Basal metabolic rate (BMR) was obtained using the Schofield equation on the basis of weight and height and according to age [68]. The BMR resulting from BIA was not used because it estimates needs with the Harris–Benedict equation, a formula not indicated for the participants of this study.

The calculation of total energy requirements (TEE) was made with (a) Culley’s equation [69], a height-based method specific for children with CP, and (b) a simplified equation [70] to calculate dietary reference standards for typically developing children, as recommended by ESPGHAN. The catch-up growth requirements were also calculated [71].

#### 2.7.5. Quantitative and Qualitative Assessment of Food Intake

Food intake registers were analyzed using PCN Pro v.1.0^®^ software according to Food Composition Tables by Farran A [72] and the Food Frequency Questionnaire through a custom-designed tool (Microsoft Access).

Caloric and protein intake was compared to recommended dietary allowances (RDAs) for age and weight [70] according to typically developing children. Carbohydrates were compared to dietary reference intakes (DRIs) for typically developing children (130 g/day) and 50–60% of total daily energy requirements. Fat intake was compared to 30% of total daily energy requirements [73]. Sugar consumption was compared to ESPGHAN recommendations of less than 5% of total daily energy requirements [74]. Fiber intake was assessed on the basis of DRI for age (25–30 g/day) [73]. Basal water requirements were assessed using the Holliday–Segar equation [75], which calculates water requirements for weight, and daily liquid recommendations through the European Food Safety Authority (EFSA) guidelines [76]. Vitamins and minerals were assessed on the basis of the Spanish Reference Dietetic Intake [76]. The dietary pattern was compared with the school healthy eating guidelines [77].

### 2.8. Statistical Analysis

Categorical variables were presented as relative and absolute frequencies and analyzed with the chi-square test or the Fisher exact test. Continuous variables were presented as mean ± standard deviation and compared with the *t*-test. For those variables that did not follow a normal distribution, we used the nonparametric Mann–Whitney U-test. Results were described and interpreted according to the obtained *p*-values. Statistical significance was accepted if *p*-values were less than 0.05. Statistical analysis was performed using GRAPHPAD PRISM 6 (San Diego, CA, USA).

## 3. Results

### 3.1. Demographic, Clinical, and Educational Characteristics of Study Group

#### 3.1.1. Health Status, Medical Conditions, Medication

Thirty-three students with severe neurological impairment with a mean age of 13.3 ± 4.9 years were included in this study, of whom 36.4% were female. Demographic and clinical characteristics are summarized in Table 1. Main diagnosis was CP in 57.6% (19), of which 18 were spastic and one was mixed type. Furthermore, 15.2% (five) had hereditary diseases (Sd. Cornelia de Lange, Sd. Smith-Lemli-Optiz, Sd. Pallister-Killian, Pompe disease, and hypermagnesemia), 12.1% (four) had epileptic encephalopathy, and 15.2% (five) had other disorders (ependymoma, meningoencephalitis), while 30.3% (10) of the study group had gastrointestinal comorbidities, 45.5% had epilepsy, and 27.3% had orthopedic disorders.

Chronic pharmacologic treatments included benzodiazepines in 51.5% of students (17), antiepileptics in 42.4% (14), and muscle relaxant compounds in 24.2% (eight). Moreover, 15.2% (five) took proton pump inhibitors, as well as vitamin and mineral supplements. Other drugs used were inhaled corticoids (12.1%), inhaled beta-adrenergic agonists (9.1%), antipsychotics (6.1%), inhaled anticholinergics (6.1%), and systemic antihistamines, opioids, and systemic anticholinergics (3%).

During the year prior to the study, 10 of these students required visits to an acute hospital emergency department. Of these, seven had to go for respiratory infection, two went for orthopedic or traumatology causes, and one went for irritability. Furthermore, eight required hospital admission, the main causes being pneumonia (three) and orthopedic or traumatology causes (three); other causes of admission were epilepsy (*n* = 1) or placement of a PEG (*n* = 1). In the previous year, the mean number of visits per student to the emergency department was 1.9, and the mean hospitalization time was 2.3 days.

#### 3.1.2. Gross Motor Function (GMFCS) and Social Factors

The assessment of the GMFCS and functional abilities described a severely impaired study group: 13 patients (39.4%) were classified as GMFCS level 5, eight (24.2%) were classified as GMFCS level 4, five (15.2%) were classified as GMFCS level 3, and seven (21.2%) were classified as GMFCS level 2. None had GMFCS level 1.

Grouping these results showed that 63.6% (21) students had GMFCS 4/5 and 36.3% (12) students had GMFCS 2/3. Those with a higher score had a greater need of PEG for feeding (38.1% (eight) vs. 16.7% (two); not significant). Following this same categorization and taking into account that the prevalence of OD was very high, it was observed that students with a lower GMFCS had a significantly lower prevalence of impaired safety of swallowing (75% (nine) vs. 100% (21); *p* = 0.040). If we analyze those who could ingest food orally, with or without PEG, individuals with GMFCS 4/5 were more dependent for eating (83.3% (10) vs. 66.7% (eight); not significant), needed more textural adaptation of the diet (83.3% (10) vs. 33.3% (four), *p* = 0.036), higher premature and uncontrolled spillage to the pharynx (83.3% (eight) vs. 16.7% (two), *p* = 0.036), and less ability to perform chewing movements (8.3% (one) vs. 41.7% (five), *p* = 0.027) than those with GMFCS 2/3.

Students and their families represented the ethnic, racial, and socioeconomic diversity of the demographics of the Maresme Region in Catalonia, Spain. Arboç school has a large range of cultural diversity (48% of the students come from different countries or have parents from countries other than Spain) and a disadvantaged socioeconomic condition (50%). The level of absenteeism at school was very high (22% in a year) mainly due to family and/or social issues related to the complex management of students, family decisions to prevent illness especially during the fall and winter seasons, and/or illness itself.

### 3.2. Oral-Motor Evaluation and Meal Observation, Swallowing Function (V-VST), EDACS, and Masticatory Capacity

#### 3.2.1. Oral-Motor Assessment Results and Meal Observation

Swallowing assessment was performed on all 32 students. According to oral-motor assessment, 81.2% (26) showed mastication impairment and 53.1% (17) had premature spillage of the bolus into the pharynx; 50% (16) of students had impaired tongue thrust, 31.2% (10) showed impaired bolus formation, and 12.5% (4) presented drooling. Up to 41.7% (10) presented spontaneous neck hyperextension when swallowing during mealtimes. The oral-motor evaluation was carried out on the 24 students who were eating by mouth. During meal observation, the most relevant results were impaired lip seal with loss of food in 54.2% (13), excessive tongue protrusion in 66.7% (16), accumulation of the bolus in the mouth in 45.8% (11), slow food management rhythm (>30 min) in 41.7% (10), and drooling in 16.7% (four). In addition, 70.8% (17) presented uncontrolled premature spillage of the bolus into the pharynx: 41.7% (10) at low viscosity (<50 mPa·s), 25% (six) at medium viscosity (250 mPa·s), and 4.2% (one) at high viscosity (800 mPa·s). Moreover, 76.5 (13) had adequate posture for feeding, 29.3 (seven) were eating with autonomy, and only one had hyperactivity. None had an aversion to food.

Regarding the feeding method used, up to 75% (24) students were fed orally and 31.3% (10) had a gastrostomy. Of the latter, 12.1% (two) combined oral feeding with PEG. Only 21.9% (seven) of evaluated students were independent feeders and the rest needed professional assistance.

#### 3.2.2. Prevalence of OD and Effect of Bolus Volume and Viscosity (V-VST)

All the students included in the SLP assessment (32) had OD; those who could be fed orally underwent oral-motor evaluation, masticatory capacity, and clinical assessment of swallowing function (V-VST) at the school. Those fed and hydrated exclusively by PEG (25% (eight)) were previously evaluated by VFS at the hospital. Considering all diagnostic methods together, up to 90.6% (29/32) had impaired safety of swallow and all of them had efficacy impairments. Twenty-four students were assessed by the V-VST, of whom 91.7% (22/24) were fed and hydrated orally and 8.33% (2/24) were fed and hydrated orally, as well as using a PEG tube. Signs of impaired efficacy and safety of swallow were found in 100% (24/24) and 87.5% (21/24) of students, respectively.

**Figure 1:**
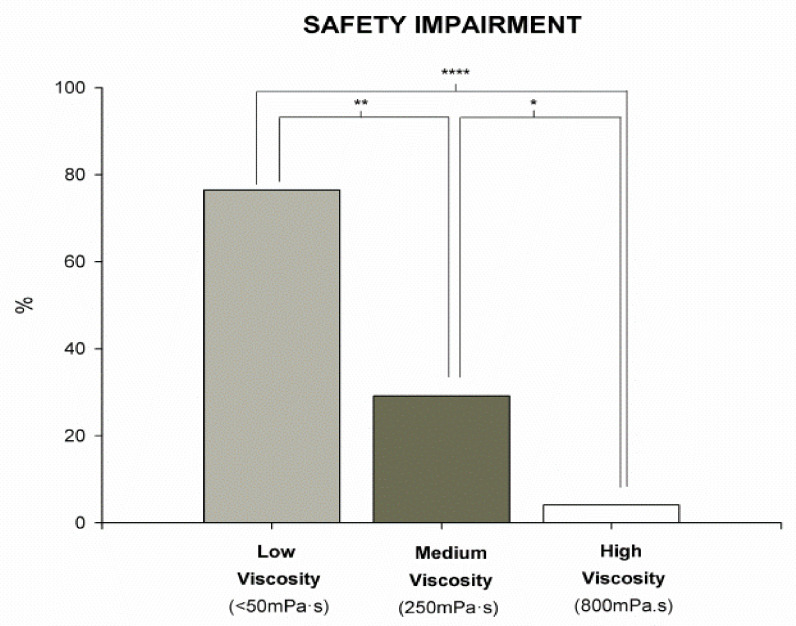
Prevalence of patients with impaired safety of swallow in the different tested viscosities (low, medium, and high) according to Volume Viscosity Swallowing Test (V-VST). * *p* < 0.05, ** *p* < 0.01, **** *p* < 0.0001.

**Figure 2:**
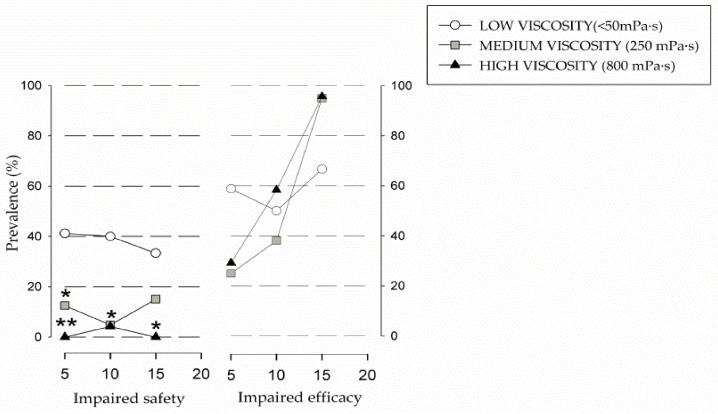
Prevalence of patients with impaired efficacy and safety of swallow according to the different levels of viscosity of the Volume Viscosity Swallowing Test (V-VST). * *p* < 0.05, ** *p* < 0.01 vs. liquid. Effect of volume on efficacy impairment GLM (*p* < 0.001 medium viscosity; *p* = 0.070 liquid; *p* < 0.001 high viscosity. Effect of volume on safety impairment GLM (*p* = 0.083 medium viscosity; *p* = 0.178 liquid; *p*—high viscosity). GLM: general linear model.

Results from the V-VST showed higher prevalence of unsafe swallows with thin liquid viscosity (76.5%) (*p* < 0.0001) vs. 250 mPa·s (70.8%) and 800 mPa·s (4.4%). The safest viscosity was 800 mPa·s, with significant differences when compared with thin liquid (4.4% vs. 76.5% unsafe swallows, *p* < 0.0001; *n* = 17) and 250 mPa·s (4.4% vs. 70.8% unsafe swallows, *p* < 0.0479; *n* = 24) (Figure 1). When we analyzed the effect of volume on the prevalence of unsafe swallows, we observed significant differences between liquid and 800 mPa·s in 5 mL, 10 mL, and 15 mL, respectively (41.2% vs. 0%, *p* < 0.0083; 40% vs. 4.2%, *p* < 0.019; 33.3% vs. 0%, *p* < 0.0369). We also found significant differences between 5 mL 250 mPa·s and thin liquid (12.5% vs. 41.2%, *p* < 0.0044). The most prevalent clinical sign of unsafe swallow was cough, observed in 54.2% (13) of the students during V-VST. (Figure 2). According to the GLM, we found a significant effect of viscosity on the prevalence of students with safe swallow (*p* = 0.004 at 5 mL, *p* = 0.035 at 10 mL, and *p* = 0.178 at 20 mL).

Regarding impaired efficacy of swallow, we found a high prevalence in all the tested viscosities: 100% at 250 mPa·s (*n* = 24), 95.8% at thin liquid viscosity (*n* = 24), and 82.4% at 800 mPa·s (*n* = 24), with no significant differences between them. It was observed that, as the volume increased with medium and high viscosity, the prevalence of impaired efficacy of swallow also increased. Low viscosity followed a similar trend except for the 10 mL volume, which had a lower prevalence than the 5 mL volume (Figure 2). The most prevalent clinical sign of impaired efficacy was oral residue, observed in 71.2% of students, followed by pharyngeal residue in 58.3% of cases. Summarizing, V-VST results showed high prevalence of clinical signs of impaired safety of swallow with thin liquids (<50 mPa·s) that significantly improved in a viscosity-dependent manner, with high viscosity (800 mPa·s) being the safest for the students, followed by medium viscosity (250 mPa·s). Additionally, increasing bolus volume and viscosity increased the prevalence of oral and pharyngeal residue. According to the results found, medium volume and viscosity (10 mL at 250 mPa·s) was both the safest and the most effective fluid recommendation for our study group.

#### 3.2.3. Eating and Drinking Classification System (EDACS)

According to the EDACS, 3.1% (one) of the students were in Level II—eating and drinking safely but with some limitations to efficiency, 65.6% (21) were in Level III—eating and drinking with some limitations to safety and limitations to efficiency, 6.3% (two) were in Level IV—eating and drinking with significant limitations to safety, and 25% (eight) of them were unable to eat and drink safely and used a PEG as the exclusive method for nutritional intake (Level V). There were no students in Level I.

#### 3.2.4. Masticatory Capacity

In addition to the items already exposed in Section 3.2.1, the masticatory assessment using a Marie biscuit showed that only 25% (six) had correct mastication movements, 33.3% (eight) had adequate lateral tongue movements, and 41.7% (10) had correct bolus handling during the oral phase of swallowing. 

### 3.3. Oral Health (OH) Status

The oral health assessment was performed on 31 students and consisted of occlusion assessment, evaluation of oral hygiene, and prevalence of periodontal diseases and caries.

#### 3.3.1. Occlusion Assessment

Out of the individuals studied, 51.6% (16) showed malocclusion according to Angle’s Malocclusion Classification, with 43.8% (seven) of individuals in Class I, 18.8% (three) in Class II, and 37.5% (six) in Class III.

#### 3.3.2. Oral Hygiene

OH was assessed according to OHI-s. The mean score was 2.06 ± 1.17 (DI 1.26 ± 0.68; CI 0.86 ± 0.6). We found that 16.1% (five), 64.5% (20), and 19.3% (six) of patients had good, fair, and poor OH, respectively. In general, we observed that a higher degree of functional disability (GMFCS) of the students correlated with a worse state of OH, although the results did not reach statistical significance (Supplementary Material S2).

#### 3.3.3. Periodontal Assessment

Up to 83.9% (26) had gingivitis; out of them, 61.5% (16) had mild, 23.1% (six) had moderate, and 15.4% (four) had advanced gingivitis. Up to 22.6% (seven) had periodontitis and all were in Stage I Grade A, according to the new classification of periodontal and peri-implant conditions (Supplementary Material S3).

### 3.4. Nutritional and Hydration Status

#### 3.4.1. Anthropometric Measurements

**Figure 3:**
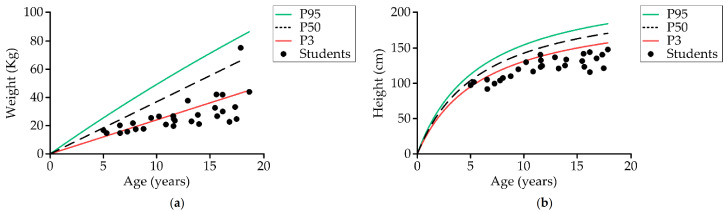
Relationship between student’s (**a**) weight and age, and (**b**) height and age compared to normal developing children reference population obtained from cross-sectional study (*n* = 29) [78]. Lower limit: weight in the third percentile; upper limit: weight in the 97th percentile. P95: 95th percentile; P50: 50th percentile; P3: third percentile.

Table 1 shows the anthropometric characteristics of the 33 students divided into two main groups of age. Comparing the weight according to age and sex with a reference population without disability [78], 69% (20) of students up to the age of 18 (29) were below the third percentile, and 96.6% (28) were below the 50th percentile (Figure 3a). Comparing the height, 89.7% (26) were below the third percentile and 100% were below the 50th percentile (Figure 3b). Body fat based on Slaughter’s equation was evaluated in 30 students. The mean percentage of fat value was 16% ± 5.7%. Comparing the total body fat with reference values in the healthy population, 74% (20) had a percentage of fat below the 50th percentile, 25.9% (seven) were in the fifth percentile, 7.4% were in the second percentile, and 18.5% (five) were lower than the second percentile. 

#### 3.4.2. Bioimpedance Results: Body Composition and Hydration Status

(a) Body Composition: Skeletal Muscle Mass, Body Fat Mass, and Fat-Free Mass

**Figure 4:**
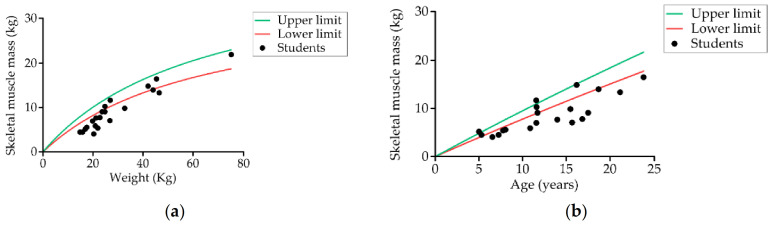
Relationship between students’ (**a**) skeletal muscle mass and weight, and (**b**) skeletal muscle mass and age compared to individual reference parameters obtained by bioelectrical impedance analysis (*n* = 20).

Table 2 shows the body composition data obtained by bioimpedance. Measurements were obtained from 60.6% (20) of students. It was not possible to take the measurement of the remainder because the electrodes could not be kept in place due to spastic movements. We found that the values of all parameters evaluated were below the normal range except for body fat, which was higher. Of the students measured (20), 85% (17) had low skeletal muscle mass (Figure 4), 55% (11) had excess body fat mass, and 10% (two) had low fat-free mass.

(b) Hydration Status: Water Compartments

**Figure 5:**
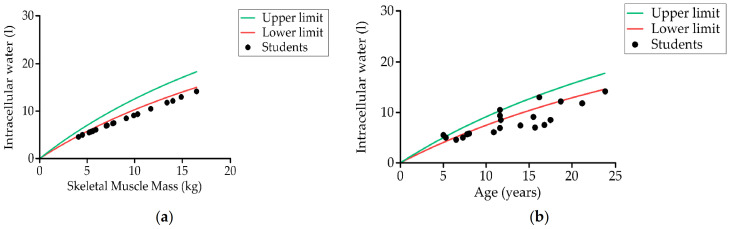
Relationship between students’ (**a**) intracellular water and skeletal muscle mass (kg), and (**b**) intracellular water and age (years) compared to reference values (*n* = 20). The lines in the graphic were obtained from the individual reference values.

Up to 70% (14) of students had dehydration in terms of intracellular water (Figure 5), 40% (eight) had dehydration in terms of extracellular water (ECW), and 60% (12) had dehydration in the whole body (total body water, TBW). No significant differences were observed between the two age groups. The ECW/TBW ratio was 0.41 ± 0.01 (0.41 ± 0.01 for the 5–12 years group and 0.41 ± 0.01 for the 13–23 years group), higher than the value expected for a healthy person (0.36–0.39). The ECW/TBW ratio in the whole body was 0.41 ± 0.01 in the group with GMFCS = V and 0.41 ± 0.007 in the group with GMFCS II–IV.

(c) Body Cell Mass and Phase Angle (PhA)

Up to 70% (14) of students had low body cell mass and the remainder (five) had normal range but very close to low range. The mean value of body cell mass was 11.74 ± 5.13 kg (normal range: 12.88–15.76). The mean PhA at 50 kHz was analyzed according to age group, degree of disability (GMFCS), and body segment. Mean PhA in the whole body was 4.305 ± 0.69 (3.945 ± 0.52 in the group of 5–12 years and 4.700 ± 0.64 in the group of 13–23 years).

#### 3.4.3. Nutritional Intake Assessment

Most families (87.9%, *n* = 29), including those with children that had feeding tubes, completed the three intake registers (24 h recall, 7 day feeding history, and Food Frequency Questionnaire); 66.6% (22) of these families completed them fully and 21.2% (seven) completed them partially, whereas 12.1% (four) did not complete them. The mean number of days evaluated in the 7 day feeding history was 4.97 ± 3.70 (5.4 for 5–12 years group and 4.6 for 13–23 years group). The meal log at the school was recorded for all students who completed the registers at home.

(a) Energy Intake

**Figure 6:**
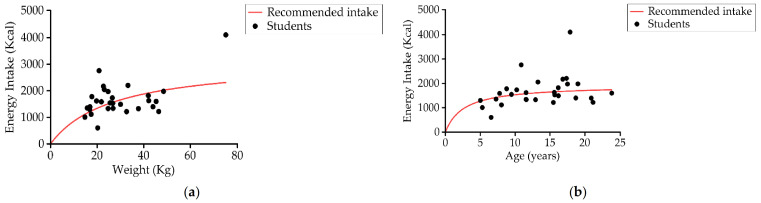
Relationship between students’ (**a**) energy intake (kcal/day) and weight, and (**b**) energy intake (kcal/day) and age compared to recommendations of energy intake (*n* = 29).

Daily energy intake was 1664.2 ± 629.7 kcal/day (1460.2 ± 484.4 kcal/day in the 5–12 years age group (*n* = 14) and 1854.6 ± 703.3 kcal/day in the 13–23 years age group (*n* = 15); 48.3% (14) had an insufficient energy intake according to the recommended dietary allowance (RDA) [70] (Figure 6).

(b) Protein Intake

**Figure 7:**
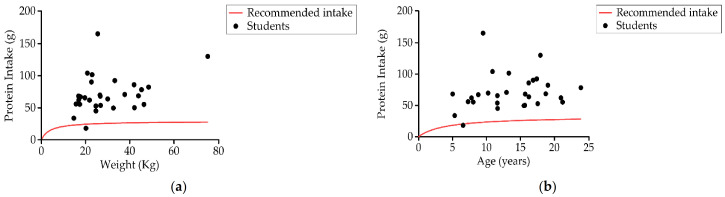
Relationship between (**a**) student protein intake (g) and weight, and (**b**) protein intake (g) and age compared to recommendations of protein intake (*n* = 29).

Up to 96.5% (28) of students consumed more protein than recommended by age and gender (Figure 7). The average protein consumption was around 2–3 g of protein per kg of body weight per day (3.1 and 2 g/kg in each group) while recommended values were 0.85 g/kg/day and 0.95 kg/day, respectively (Table 3). The contribution of proteins to the total daily caloric percentage was 17.4% ± 5.6% (18.1% ± 7.6% Group 1 and 16.7% ± 2.8% Group 2).

(c) Water Intake

**Figure 8:**
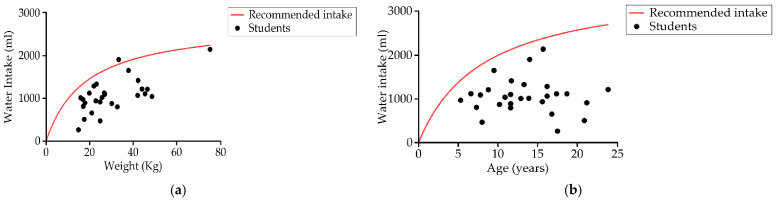
Relationship between (**a**) student water intake and weight, and (**b**) water intake and age compared to basal liquid recommendations [75] and liquid recommendations (European Food Safety Authority, EFSA) respectively [80] (*n* = 28).

Up to 96.4% (27) of the students had recordings below the daily basal recommendations of fluids [75] (Figure 8a), and all the students’ results were below the daily liquid recommendations for their age (Figure 8b). Mean water intake, from both drink and food origin, was 1034.6 ± 437.6 mL, less than half the recommended liquid intake for this study group (Table 4) [80].

(d) Other Macronutrients, Micronutrients, and Fiber Intake

Carbohydrate intake was 198.2 ± 85.7 g/day, representing 47.5% ± 7.5% of total daily energy. Sugar consumption was 80.6 ± 65.9 g/day (19.4%), over the maximum 5% of total daily energy intake recommended. Statistically, a significant difference was obtained between the two age groups in sugar consumption (54.7 ± 32.9 g/day and 104.8 ± 79.9 g/day, *p* = 0.0240). Fat intake was within the parameters of normality (64.4 ± 30.9 g/day, which represented 34.6% ± 7.5% of total daily energy intake. Fiber intake was 16.2 ± 8.4 g/day (13.4 ± 6.9 and 18.9 ± 9 g/day in each group) far below the recommended 25–30 g. Both groups presented a low intake of most minerals and vitamins compared with recommendations for healthy children. The mineral deficiencies observed in both groups were calcium, magnesium, zinc, sodium, and potassium, while the vitamin deficiencies were vitamins B2, B3, B6, B9, D, and E. With regard to iron, phosphorus, and vitamins A, E, and B1, differences between the two groups was observed. Only vitamin B12 and vitamin C were covered by both groups (for specific values, see the table in Supplementary Materials S4).

#### 3.4.4. Food Intake Assessment Results and Dietary Pattern

Dietary information of 21 students was obtained using the Food Frequency Questionnaire. The missing 12 students did not complete or return this questionnaire.

Protein intake was very high, especially proteins of animal origin (mainly meat and dairy products), whereas it was very low in products of vegetable origin. According to the Guide to Healthy Eating at School [77], around 40% of the students ate twice the recommended amount of processed meat (sausage, ham, etc.). In contrast, consumption of fish and eggs was low; 75% ate fewer than three eggs per week and 40% ate fewer than three portions of fish per week. Furthermore, 95% of the students ate fewer than 3–6 portions of nuts per week and 85% ate fewer than 3–4 portions of legumes per week. High complex carbohydrate food consumption was also poor; 85% ate fewer than three portions per day of farinaceous food, and those that did ate mainly potatoes and cereals in refined form (not whole). Their vegetable consumption (mainly cooked) was higher than their fruit consumption; 70% ate cooked vegetables daily but no student ate more than one portion of raw vegetables per day. Only 10% ate three or more portions of fruit a day. For cooking and dressings, olive oil was the most consumed fat; 90% consumed more than six tablespoons of oil per day. The consumption of sugar products was very high; 80% ate sweetened products, 55% drank juices and soft drinks, 85% ate sweetened dairy desserts, and 75% ate cookies or cereal bars. Water consumption was very low; 90% had an average consumption of four glasses per day. Tea, coffee, and infusions were drunk by 40% of the adolescent group, mainly by those students of Moroccan descent.

#### 3.4.5. Prevalence and Severity of Malnutrition

Prevalence and severity of MN depend on the criteria used to establish the diagnosis. According to WHO Growth Standards, the prevalence of chronic MN (HAZ <−2) was 89.3%, with 75% being severely malnourished (stunted). Prevalence of acute MN (BAC <2) was 21.4%, with half of them being severe, and the prevalence of overall MN (WAZ <2) was 55.5%, with 33.3% of them being severe. The prevalence of overweight and obese students was 21.4%. 

According to the Waterlow Index, 96.4% of students had chronic MN (WI for height), with 57.1% of them being severe; 17.8% had acute MN (WI for weight), with 7.1% of them being severe (Table 5). The average value of Waterlow for height was 84.2% ± 6.1% (severely malnourished), and the average value of WI for weight was 102.6% ± 22.5% (normal nutritional status). There was a significant difference in chronic MN between the two age groups, with the older age group being more pronounced (80% ± 5.2% vs. 87.4% ± 4.8%, *p* = 0.002). 

According to these growth charts from children with disabilities based on GMFCS, the prevalence of risk of MN was 30.30% (10), and that of MN (BMI ≤10th percentile) was 12% (four). The results obtained with these curves certainly show a much lower prevalence of underweight students compared to the curves for normal developing children. 

#### 3.4.6. Energy Requirements

**Figure 9:**
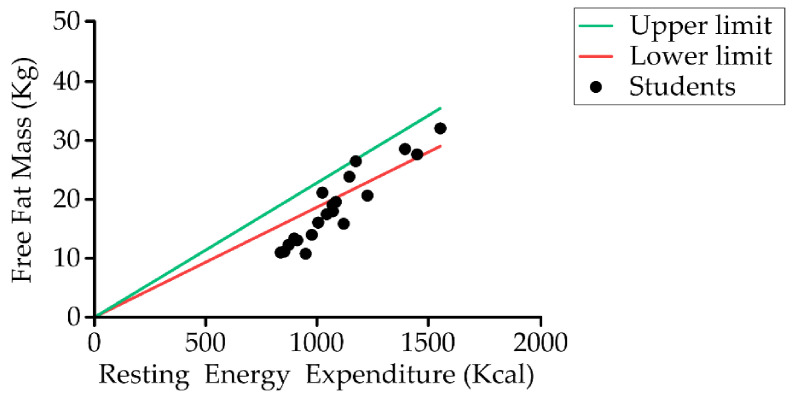
Relationship between student resting energy expenditure (Schofield) and fat-free mass (kg) (*n* = 20).

The basal metabolic rate (BMR) obtained using the Schofiel equation was 1082 kcal/day (977 kcal/day for Group 5–12 years and 1187 kcal/day for Group 13–23 years, *p* < 0.01). Up to 60% (12) were below the BMR (Figure 9). According to Culley’s equations, the total energy requirements (TEE) were 1539 kcal/day, a value similar to that obtained by means of simplified equations (1589 kcal/day). There were significant differences between the two age groups for both BMR and TEE. The catch-up growth requirements estimated were around 1784 kcal (1554 kcal/day and 1846 kcal/day) (Table 6).

## 4. Discussion

The main results of this study showed that children from L’Arboç SNS presented the following characteristics: all of them had swallowing disorders, 90.6% with impaired safety of swallow; 96.9% had eating and drinking disabilities according to EDACS (68.7 with II-III and 31.3% with IV–V); 83.9% had poor or fair OH; 89.3% and 21.4% had chronic and acute MN, respectively; 70% had dehydration. In addition, they had a wide age range and several types and severity levels of comorbidities, polymedication, and physical and intellectual disabilities. They were frail and their clinical outcomes were poor with high rates of emergency room (ER) visits and hospitalizations. Their educational and social outcomes were also poor, with a high level of absenteeism. 

The Arboç school students had severe neurological conditions and a very high level of physical impairment according to GMFCS (63.6% level IV–V). Spastic CP was the most frequent neurological condition, and epilepsy and chronic gastrointestinal impairments were the most common comorbidities [82]. These high levels of health frailty lead to frequent hospital and ER admissions, as well as multiple visits to medical specialists, with respiratory infections being the main cause of ER admissions. Our students were polymedicated, with a high percentage of consumption of benzodiazepines, antiepileptics, and other muscle relaxants. Although the purpose of the school is educational, the care needs of the students require adapting the day-to-day life of school attendance to circumstances such as high absenteeism, which makes school health monitoring even more complex (Supplementary Material S5).

Regarding swallowing function, we found that OD was extremely prevalent and highly severe in our SNS students according to the oral-motor, EDACS, and swallowing assessments. All study participants had OD with a high prevalence of impaired safety of swallow. Similar results were obtained by Calis in a study of 166 children with neurological impairment (NI), where the prevalence of OD was 99% [3,32]. The proportion of students fed by gastrostomy in our study (31.3%, 25% exclusively and 6.3% mixed) was greater than that obtained by other authors. Caramico et al. noted 17.5% (12.5% exclusively and 5% mixed) after assessing 40 children with CP [83], and Dahlseng described gastrostomies at 11% [34]. However, when Dahlseng stratified the CP patients according to GMFCS (Level IV–V), the prevalence was 32%, which coincides with the numbers in our study. All these data further confirm the close correlation between GMFCS and OD, which is also well defined in the literature [84].

Disruption of the oral phase leads to alterations in both the efficacy and the safety of swallowing. High rates of oral phase impairments (66.7% tongue protrusion, 54.2% incomplete lip seal, and 45.8% accumulation of residue in the mouth) were observed in Arboç students, which can lead to feeding difficulties and MN. Other authors described chewing impairment (21%), motor speech articulation (36%), and oral-motor delays (44–47%) [84]. Cervical hyperextension was described in 41.7% of Arboç students, and Furkim found this characteristic in more than 50% of individuals with CP [85]. This last condition, together with an ineffective velopharyngeal seal, can lead to aspiration because it favors premature spillage of the bolus into the pharynx while the pharynx is still in a respiratory configuration. Up to 90.6% (29) of the study sample had signs of impaired safety of swallow and aspiration, which have been associated with poor long-term prognosis [86,87]. Benfer noted that the most common signs on direct assessment were cough (44.7%), multiple swallows (25.2%), gurgly voice (20.3%), wet breathing (18.7%), and gagging (11.4%). Furkim also found suggestive signs of aspiration during clinical evaluation, especially with liquids [85]. It should be noted that OD is frequently a neglected condition, and many of these students are not diagnosed in SNS where there may be low awareness of OD. Compensatory strategies to ameliorate these findings, such as increasing bolus viscosity, have been proven to be a valid strategy in several phenotypes of dysphagic patients [88]. Our results showed that the safest viscosity was the highest (800 mPa·s) and the least safe was liquid (<50 mPa·s). These results are in line with our previous studies which showed the strong therapeutic effect of increasing viscosity up to level of 800 mPa·s [48]. A similar effect was also described in neurodegenerative diseases and stroke patients [89]. We also observed that our students presented a high prevalence of efficacy impairment, particularly oral residue (95.8%) and pharyngeal residue (83.3%), which directly correlated with an increment in volume and viscosity. In this specific study, 250 mPa·s and 10 mL were selected as the most effective viscosity and volume for thickened fluids.

One of the main risk factors for the development of respiratory complications in patients with OD is poor oral health and colonization by respiratory pathogens [20]. In our study, we found similar results to those found by other authors and confirm the low awareness of this condition in this specific phenotype. We observed that 51.6% of students showed malocclusion, 83.9% had gingivitis, and 22.6% had periodontitis. Orellana and collaborators, in a study on CP, reported a higher prevalence of dental malocclusion (84%) and similar results regarding gingivitis and periodontal disease (67% and 14%, respectively) [90]. Up to 83.9% of our students needed to improve their OH, with 64.5% of them being in fair status and 19.4% in poor status. Orellana also described poor OH since the total of the study group presented more than 60% plaque and 50% calculus on all tooth surfaces. In its 2020 annual report, the Spanish Association of Dentists reported a prevalence of 40.8% in healthy young people (12–15 years) with a healthy periodontium [91]. Students’ caregivers, usually parents, reported that the main problem was rejection of toothbrushing, which often made it impossible for them to perform the technique correctly, a justification of the situation. It is well known that two of the main complications of OD are respiratory infections and aspiration pneumonia [92]. We previously found that frail older patients with OD had poor OH, high oral bacterial load, and prevalence of oral colonization by respiratory pathogens, and they presented high risk for respiratory infections [50,93]. During the previous year, one-third of Arboç students had a respiratory infection, and three of them were hospitalized with pneumonia. We previously found that OD, poor oral health, and colonization by respiratory pathogens and MN are risk factors associated with respiratory infections, including aspiration pneumonia [4,94]. On the other hand, we previously proved that interventions with the aim of treating these main risk factors (dysphagia with fluid adaptation, malnutrition and vulnerability with texture adaptation and nutritional supplementation, and impaired oral health and hygiene to reduce the bacterial load of the oral cavity) are effective in reducing the incidence of respiratory infections and improving nutritional and clinical outcomes in older patients with OD [51]. Therefore, we aim to apply these strategies in L’Arboç students in future studies.

Nutritional status is also a key factor for the development of complications, and it is associated with OD. Poor nutrition and growth impairments are common findings in children with CP. Studies showed that these individuals are shorter and thinner than similarly aged healthy children [33,95,96], and that they have difficulties in growing. This was fully confirmed in our study where, regarding weight, 96.6% of study participants were below the 50th percentile and 69% were below the third percentile; regarding height, 89.7% were below the third percentile, while none of our students were above the 50th percentile. Main factors causing low weight and height in this group are nutritional but also brain damage and the type, distribution, and severity of the motor impairment, as one Spanish study found significantly lower body weight in the group with the highest GMFCS [32]. CP-specific growth charts describe substandard growth as they include many children with other health conditions that affect growth, particularly malnutrition. ESPGHAN does not recommend using these charts as they do not measure how this group of children should ideally grow [32]. Our main results were obtained by comparison with standard growth charts validated by ESPGHAN [32]. Studies have also reported that the intake of energy and nutrients in this group is considerably lower than the recommended daily allowances [26,97]. Caramico et al. found that patients with CP and OD received lower daily energy [83]. However, another study based on preschool children with CP found that energy requirements in ambulant children were similar to healthy children [13]. Other studies conducted in Norway found that the presence of orofacial dysfunction was associated with reduced daily energy intake [98]. Difficulty biting (70%), cleaning behaviors (70%), and chewing (65%) were the most common deficiencies in processing solid foods in CP [99]. In our study, we found high prevalence of impaired chewing and swallowing in students (66.7% tongue protrusion, 54.2% incomplete lip seal, 45.8% accumulation of bolus in the mouth, and 100% of students with efficacy impairments of swallow, the main cause of insufficient nutrient intake). Moreover, the texturization that these children received before the study was suboptimal, which made it even more difficult to meet their caloric and nutritional requirements.

Differences in energy expenditure also play an important role in children with CP. Growth failure in these children also depends on non-nutrition-related factors such as the severity and type of neurological impairment, mobility, and cognitive function [100]. Increased muscle tone, level of physical activity, and the presence of involuntary movements may also contribute to increased daily energy expenditure in CP [101]. However, several studies have confirmed that the main cause of growth failure and malnutrition in children with neurological dysfunction is insufficient caloric and protein intake [102,103,104]. In our scenario, around 50% of students presented an insufficient energy intake according to recommendations for weight and age. As expected, the students at our school had low fat-free mass, low skeletal muscle mass, and low cell mass, results that are consistent with most studies [96,104]. However, some students at Arboç had excess fat mass, both in quantity and percentage. This finding is consistent with Romano [105], who noted that children with marked NI had high fat mass compared with reference children of similar age and sex. Metabolic principles describe that, if nutrient intake is insufficient to meet the needs, resulting in malnutrition, body fat and muscle will be catabolized to provide energy [106]; thus, it could be expected that fat would also be low in our students. Increased risk of body fat accumulation may be associated with insufficient physical activity in children with NI and with the high consumption of sugar observed in the intake records, which are associated with increased risk for obesity [107]. Another important finding of our study is the low body cell mass value, which is considered an important indicator of nutritional status in this group and inversely related to the degree of GMFCS [108].

The energy distribution from the daily intake of macronutrients we found in our study (47.5% carbohydrate, 34.6% fat, and 17.4% protein) was similar to that found in another study [109]. In general, students ate excessive amounts of protein and insufficient complex carbohydrates. Protein intake was very high (≥2.5 times the RDA), especially in the younger group (5–12 years), which also agrees with other authors [83,97]. Excess of protein intake could be due to the belief of families and caregivers that protein intake alone can improve muscle mass, but evidence shows that it is necessary to combine nutrition with exercise to maintain muscle function [110]. Protein requirements of the students differ depending on whether they are established on the basis of the current weight of the children [79] or the general recommendations for healthy children (30–54 g/day) [76]. Neither result is compatible with the protein requirements in healthy patients with an optimal weight according to age. Most of them have insufficient current weight, and these optimal amounts are excessive for them. Therefore, it is important to be careful and avoid daily protein excess. The same occurs with energy intake, calculated from the current weight of the students (Group 1: 1460.2 ± 484.4 kcal and Group 2: 1854.6 ± 703.3 kcal), whereby they establish needs that are underestimated in relation to what they theoretically need by age (1700–3000 kcal) [79]. Taking this into account, we observed that the intake of Group 1 coincided with that estimated according to weight, but not with that estimated by age. Group 2, however, exceeded the caloric intake according to weight, but would not cover the needs according to age. In order to improve the food offered in the school canteen, our group established guidelines on calorie and protein recommendations for the two school groups. Regarding carbohydrates (CH), the students met the recommended minimum of 130 g/day; however, they did not meet the percentage of the total recommended daily caloric intake (50%), which does not favor muscle synthesis or weight gain of the students. Caramico et al. also noted that patients with OD received lower amounts of CH (median: 170.9 g vs. 234.5 g, *p* = 0.023). One possible reason for the low intake of complex CH could be the difficulty in providing complex CH in blended diets for people with OD and CP [83]. On the other hand, consumption of free sugar exceeded the recommended 5% of the total daily caloric intake in both groups [74] (15% in Group 1 and 22.6% in Group 2), which could lead to an increased presence of caries [111] and possible alterations in body composition, thereby increasing body fat deposits, as we observed in our results.

We also observed an imbalance in the lipid profile of the diet, with a low contribution of unsaturated vs. saturated fats, resulting from the high consumption of meat and processed meat. Poor intake of fiber (16.2 vs. 25–30 g/day) was observed in both age groups of our students, as also found by some authors [112,113]. Low dietary fiber intake combined with low fluid intake may also contribute to the development of constipation. In general, both groups presented a low intake of most minerals and water-soluble and fat-soluble vitamins, possibly due to an unbalanced diet. The nutritional results correlate with the qualitative evaluation of the diet: a low intake of fiber and folic acid possibly explained by the insufficient intake of fruits, vegetables, legumes, and nuts, as well as an insufficient intake of calcium and vitamin D, both with an important role in bone formation/growth, due to an insufficient intake of dairy products, legumes, and nuts. The high consumption of meat products allows them to cover their iron and vitamin B12 requirements; however, it does not cover the recommended intake of zinc due to the low consumption of seafood, legumes, whole grains, and nuts. This qualitative and quantitative relationship indicates that, beyond digestive problems that may reduce nutrient absorption or drug–food interactions, a balanced diet can help improve nutritional intakes in these SNS students. The students had inappropriate dietary patterns. The Mediterranean food pattern is characterized by grains and vegetable bases with meat or similar as a “garnish” and type of fat (olive oil, fish, and nuts), rich in micronutrients provided by seasonal vegetables, herbs, and spices [114]. The students’ diet was highly unbalanced, due to the low content of vegetables and excessive content of meat and sweetened products. Low intake of fruits and vegetables (sources of dietary fiber) was previously seen in another study developed in children with CP [115]. Furthermore, low consumption of fish and no consumption of nuts was also observed. To conclude, the three main objectives for intake improvement are (1) to increase caloric intake by increasing the consumption of complex carbohydrates, (2) to improve the lipid profile of the diet, increasing the consumption of monounsaturated and polyunsaturated fats, and promoting the consumption of vegetable versus animal protein, and (3) to ensure the requirements of micronutrients by consuming fresh, whole-grain, and vegetable products.

Nutritional evaluation, according to WHO Growth Standards [116], also showed that stunting (chronic MN) was the most common form of MN (89.3%) in our students, followed by underweight (overall MN, 55.5%) and thinness (acute MN, 21.4%). We can highlight two important aspects of our results. First, the prevalence of MN in the older group was higher than in the younger (87.4 vs 80%, *p* = 0.002); second, the prevalence and severity of chronic MN were higher in our study than observed by other authors [23,117,118,119,120]. Two possible reasons can justify this higher severity: the higher age and high degree of disability of our study group population. Previous studies suggested that prevalence of MN in CP increases with age. Karim et al. found a prevalence of MN of 29% in children aged 5.6 years, and Almunet et al. determined a prevalence of 50% for MN [117] in children aged 7 years [14,23]. The fact that our study group population included students between the ages of 4 and 23 may have been one of the reasons for our higher prevalence of MN. The second reason could have been the higher degree of disability in our study group population. It is known that a greater degree of disability results in a worse nutritional status [121]. In our study, 63.6% of students had GMFCS IV–V, and 75% of the stunted group population was severe, whereas Jahan found this degree of disability in less than 50% and severe stunting in 52.4% [23]. The prevalence of acute MN we found in our study coincides with that found by Bell et al. (23%) (6 years, *n* = 89) [118]. Furthermore, the prevalence of overweight and obesity we found (21.4%) is in complete agreement with that found by Martinez de Zabarte [122]. Another tool we used to measure MN was the Waterlow Index, a method widely used in clinical practice, obtaining prevalence values similar to those obtained using the *z*-score: 17.8% vs. 21.4% in acute MN and 96.4 vs 89.3% in chronic MN. We, therefore, consider the WI a good tool for classifying MN in SNS. In addition, all the above information was obtained by comparing with standard growth charts validated by ESPGHAN for children with CP [32]. However, it should be noted that their growth patterns are often notably different from healthy children. Nevertheless, CP-specific growth charts describe growth which is not necessarily ideal, as they also consider many children with health issues affecting growth, especially MN; for this reason, they are not recommended by ESPGHAN [32].

Estimation of energy requirements in children with CP is the first step toward a personalized nutritional intervention. In our study, resting energy expenditure (REE) of students was similar to data reported by Bell et al. in children with bilateral spastic CP aged 5–12 years who found a resting energy expenditure of 1074 ± 168 kcal/day and total energy expenditure of 1674 ± kcal/day [118]. Other authors reported lower resting energy expenditure (between 760 and 876 kcal/day) [123] and total energy expenditure of 1367 ± 329.17 kcal [95], both in younger populations. Several publications have suggested that children with bilateral spastic CP have the highest resting energy expenditure and total energy expenditure (due to the hypertonicity of the muscles), whereas children with spastic quadriplegic CP have the lowest [26,95,123,124,125,126,127]. Studies of Walker and Rieken showed a decline in the total energy expenditure with increasing GMFCS level [95,128]. Similar results were observed when considering the number of limbs involved [95]. A trend toward lower energy requirements was observed when the number of limbs involved increased. Further research is needed in order to determine the influence of motor type on energy requirements. Moreover, other factors which influence energy requirements that need to be taken into account are level of physical activity, altered body composition, and MN [105]. For future studies, we could include the assessment of muscle tone (described by the Ashworth Scale) in assessing the nutritional status of children with CP to better adjust for the energy needs of children [129].

Lastly, we found a high percentage of dehydration among Arboç school students. The high prevalence of OD in our study led up to 96.4% of our students to consume below the daily basal recommendations of fluids, causing dehydration in the intracellular compartment water in 70% (14). Other studies have also reported that people with NI are at a higher risk for dehydration [130,131]. Deficiency of body water due to OD and low intake causes dehydration, which might also contribute to impaired cognitive function [132,133]. Dehydration in children and young people is associated with reduced cognitive response, while correct hydration has a positive effect on cognitive function [133]. In our study, the prevalence of dehydration was very high (70%) and mainly hypertonic. The swallowing disorders of these individuals caused low water intake, which was also associated with the difficulties encountered by caregivers to ensure safe intake. Studies also found a relationship between the state of hydropenia and oral-motor impairment of individuals [134], as well as greater dehydration in subjects with ND compared to a typical development child [130,131].

Regarding bolus viscosity descriptors, we did not use the IDDSI descriptors. Firstly, for texture-modified foods, we used the levels proposed by the British Dietetic Association (BDA) descriptors [45]: (a) thick purée (C) and (b) fork-mashable (E), due to the fact that this classification of texture modified foods is well implemented in our institution and easy to implement in the school. Secondly, for thickened fluids, we expressed the viscosity in SI units (mPa·s) as recommended in a recent paper endorsed by 11 scientific societies [135].

Our study presents many limitations. The main one was the relatively small sample size (*n* = 33) and the diverse levels of disability and socioeconomic status of our study group. Nevertheless, the sample is representative of the population of an SNS, and our results were statistically significant. It would be interesting to expand the scope of this investigation to additional studies with other SNS and a larger group of children with CP, as well as to perform the same assessment on these same students after a period of treatment in terms of OD, MN, and OH. Another limitation is that we used some evaluation tools such as EDACS and GMFCS that are only validated for CP; although this pathology was the most prevalent in our study, we had students with nine different types of neurological disorders. We used these tools as there are no specific classification systems validated for the other neurological diseases and because these tools are the ones recommended in SNS in Spain. However, we recognize that different pediatric diseases or conditions might show different types of dysphagia. A further limitation of our study is the method we used to assess mastication. Due to the severity of the impairment of the participants, it was not possible to use a validated and quantitative method such as TOMASS on all students, as most of the participants were not able to manage a biscuit. Likewise, the evaluation of drooling was assessed in a dichotomic way (presence/absence); the use of more complete indices such as the Blasco index is proposed for future studies [136,137]. Lastly, we used the V-VST to clinically assess swallowing impairments on safety and efficacy of swallow, as done in other groups with children; despite its good psychometric properties, the fact that we developed the test, and the fact that we have wide experience with it, we acknowledge that the V-VST has not yet been appropriately validated for children and, hence, its results should be interpreted with caution. We plan to develop specific studies to validate the test in this specific population.

To sum up, our results suggest that (1) a high prevalence of OD for fluids is associated with dehydration, (2) OD for fluids and solids and poor OH could be related to respiratory infections and MN, and (3) MN could be related to poor physical and intellectual development in these children. Due to the presence of OD, impaired OH, chronic MN, and intracellular DH, these children with severe conditions such as CP and ND require complex nutritional and health management. Thus, these students must be specifically managed through nutritional and educational strategies, and the multidisciplinary team at SNS should include healthcare professionals specifically trained in these conditions. Optimal management of these SNS students may improve clinical outcomes and quality of life and reduce their needs for medical care. In accordance, we are currently introducing a personalized intervention at school and at home (Supplementary Material S6). The optimal intervention consists of four steps: (a) measuring the nutritional, hydration, and OH status of all the students at the end of each school year; (b) establishing an educational program for families and informal caregivers and school staff on the management of OD, MN, DH, and OH of students (already started) (Supplementary Material S7–S9); (c) developing and implementing an hydration program using homogeneous (xanthan gum) fluid thickeners for all students and specific cups to guarantee the appropriate and safe fluid provision at the appropriate bolus volume and viscosity according to the V-VST (250 vs. 800 mPa·s) and with specific support staff for hydration (Supplementary Material 10); (d) introducing the triple adaptation of solid foods with a design (parameterization and industrialization) of fifth range dishes that meet the triple adaptation of the diet (already started)—two levels of texture (fork-mashable and purée), four levels of caloric and protein intake according to age and GMFS (1600 kcal and 30–40 g protein for 5–12 years and GMFCS II–IV; 1400 kcal and 20–25 g of protein for 5–12 years and GMFCS V; 2000 kcal and 50 g of protein for 13–23 years and GMFCS II–IV; 1700 kcal and 30–40 g of protein for 13–23 years and GMFCS V). These guidelines of textures and calories and proteins can serve as a basis for the catering companies to produce meals adapted to the special needs of these students. The final goal is to measure the effectiveness of the interventions at the end of each school year.

## 5. Conclusions

To conclude, OD, MN, DH, and poor OH are highly prevalent in SNS and are associated with poor clinical and educational outcomes. Our results highlight the unmet need of specific nutritional and educational strategies in SNS and a multidisciplinary team also skilled in the management of these issues, to promote the conditions for optimal physical, cognitive, and social development of these students. Our data clearly suggest that an improved management of these clinical situations prevalent in SNS may have an impact on clinical improvement, resulting in less need for medical care and better quality of life. 

## Figures and Tables

**Table 1:** Anthropometric characteristics of students according to age group.

Variable, Mean (SD)	Total (*n* = 33)	Group 5–12 years (*n* = 16)	Group 13–23 years (*n* = 17)	*p*
Mean age, years	13.3 (4.9)	9.1 (3.8)	17.2 (2.8)	-
GMFCS (Median IQ range)	5 (2–5)	4 (3–5)	4.5 (2–5)	0.806
II	21.2	12.5	29.4	0.235
III	15.2	25	5.9	
IV	24.2	31.3	17.7	
V	39.4	31.3	47.1	
Mean body weight, kg	28.8 (13)	21.6 (6)	35.5 (14.3)	0.001
Mean height, cm	127.2 (19.5)	115.3 (15.1)	138.4 (16.7)	0.001
Mean knee length, cm	38.5 (7.2)	34.1 (5.8)	42.7 (5.7)	0.001
BMI mean, kg/m^2^	17 (4)	16.1 (2.2)	18.1 (5.1)	0.195
MUAC, cm	20.4 (4.2)	20 (4)	20.8 (4.4)	0.3
Skinfold measurements, mm				
Subscapular s.	9.1 (7.4)	7.5 (5.4)	10.8 (9)	0.066
Triceps s.	9.3 (5.3)	9.1 (3.6)	9.5 (6.6)	0.439
Abdominal s.	7.4 (7)	6.1 (4.4)	8.8 (8.8)	0.649

GMFCS: gross motor function classification system; MUAC: mid upper arm circumference; BMI: body mass index; s: skinfolds.

**Table 2:** Body composition calculated by bioelectrical impedance analysis (BIA) (*n* = 20).

	Total Sample (SD)	Normal Range of Overall Population *
Total body water, %	52.3 (7.4)	55.8–68.2
Extracellular water, %	20.9 (3.1)	21.2–26
Intracellular water, %	31.4 (4.4)	34.6–42.3
Soft lean mass, kg	17.4 (6.1)	18.6–22.8
Soft lean mass, %	66.7 (9.4)	71.6–87.6
Fat free mass, kg	18.6 (6.4)	19.8–24.1
Fat free mass, %	71.7 (10.1)	75.9–92.8
Skeletal muscle mass, kg	8.7 (3.7)	9.9–12.2
Skeletal muscle mass, %	32.5 (5.7)	37.6–46
Cell mass, kg	11.7 (4.1)	12.9–15.8
Cell mass, %	45 (6.3)	49.5–60.6
Fat mass, kg	7.8 (5)	3.6–6.9
Fat mass, %	28.3 (10.2)	13.4–26.3

* Normal range of overall population obtained from the mean of the normality intervals according to their age provided by the bioelectrical impedance analysis for each student.

**Table 3:** Comparison of the daily protein intake of the students with the recommended dietary allowance (RDA) according to age group, mean (SD).

	Total (*n* = 29)		Group 5–12 years (*n* = 13)		Group 13–23 years (*n* = 16)	
	g/day	g/kg/day	g/day	g/kg/day	g/day	g/kg/day
Protein intake	71.2 (28.7)	2.47	66.7 (34.5)	3.1	75.4 (22.3)	2.1
RDA (for students’ weight) [79]	26.4 (11.5)	0.9	20.5 (5.7)	0.95	31.9 (12.9)	0.85

RDA: recommended dietary allowance.

**Table 4:** Assessment of water intake with basal needs and daily fluid recommendations, mean (SD).

	Total	Group 5–12 years	Group 13–23 years
Water intake (mL) of the students (including food)	1034.6 (437.6)	862.9 (436.6)	1195 (385.8)
Basal liquids (mL) requirements [75]	1652.2 (286.7)	1489.9 (167.6)	1804.9 (324.4)
Recommended daily water intake (mL) (including food) [80]	2181.8 (370)	1600–2100	2100–2500

**Table 5:** The nutritional status according to the *z*-score of WHO Growth Standards (WAZ, BAZ, and HAZ) [81] and Waterlow Index in age at enrollment.

Nutritional Status Assessment	Total, % (*n*)	Group 5–12 years, % (*n*)	Group 13–23 years, % (*n*)	*p*
WHO growth standards				
Weight-for-age *z*-score, WAZ (Overall NS), *n* = 9				
Normal	44.4 (4)	44.4 (4)	-	
Underweight	55.5 (5)	55.5 (5)		
Moderate	22.2 (2)	22.2 (2)	-	
Severe	33.3 (3)	33.3 (3)	-	
Height-for-age *z*-score, HAZ (Chronic NS), *n* = 28				
Normal	10.7 (3)	18.8 (3)	0	0.17
Undernutrition (stunting)	89.3 (25)	81.3 (13)	100 (12)	
Moderate	14.3 (4)	18.8 (3)	8.3 (1)	0.593
Severe	75 (21)	62.5 (10)	91.7 (11)	
BMI-for-age *z*-score, BAZ (Acute NS), *n* = 28				
Overnutrition (overweight + obesity)	21.4 (6)	31.3 (5)	8.3 (1)	0.382
Normal	57.1 (16)	50 (8)	66.7 (8)	
Undernutrition (thinness)	21.4 (6)	18.8 (3)	25 (3)	
Moderate	10.7 (3)	12.5 (2)	8.3 (1)	1
Severe	10.7 (3)	6.3 (1)	16.7 (2)	
Waterlow Index				
WI for weight (Acute MN), *n* = 28				
Normal	82.1 (23)	83.3 (10)	81.3 (13)	0.411
Malnourished (wasting)	17.8 (5)	16.6 (2)	18.8 (3)	
Mild	3.6 (1)	8.3 (1)	0	0.233
Moderate	7.1 (2)	8.3 (1)	6.3 (1)	
Severe	7.1 (2)	0	12.5 (2)	
WI for height (Chronic MN), *n* = 28				
Normal	3.6 (1)	8.3 (1)	0	0.504
Malnourished (stunting)	96.4 (27)	91.7 (15)	100 (12)	
Mild	17.9 (5)	25 (3)	12.5 (2)	0.614
Moderate	21.4 (6)	16.7 (2)	25 (4)	
Severe	57.1 (16)	50 (6)	62.5 (10)	

WAZ: weight-for-age *z*-score (for 5–10 years); HAZ: height-for-age *z*-score (for 5–19 years); BAZ: BMI-for-age *z*-score (for 5–19 years); NS: nutritional status; MN: malnutrition; WI: Waterlow Index.

**Table 6:** Description of energy expenditure and energy requirements for growth recovery comparison and comparison by age, mean (SD).

	Total, SD (*n* = 33)	Group 5–12 years (*n* = 16)	Group 13–23 years (*n* = 17)	*p*
BMR, kcal/day				
Schofield equation	1081.89 (186.2)	977 (127.7)	1186.79 (178.4)	0.0005
TEE, kcal/day				
Culley equation	1538.61 (301.3)	1400.37 (239.3)	1676.86 (299.2)	0.0065
Simplified equations based on weight [68]	1589 (425)	1459.4 (295)	1488.1 (542.5)	0.8530
Catch-up growth requirements, kcal/day				
kcal/day	1783.74 (228.8)	1554.62 (101.89)	1847.06 (227.71)	0.0005
kcal/kg/day	62 (17.60)	71.94 (13.25)	52.03 (15.88)	
Mean body weight (kg)	28.77 (13)	21.61 (7.69)	35.50 (14.34)	

BMR: basal metabolic rate; TEE: total energy requirements.

## Supplementary Materials

The following are available online at https://www.mdpi.com/article/10.3390/nu13072413/s1; S1: A further explanation of the Rationale, Hypothesis and Aim of the Study in the context of the Program for Management of Malnutrition and Swallowing Disorders at l’Arboç School; S2: Access to educational materials with more than 100 video recipes of the more than 250 recipes, adapted according to the degree of dysphagia and the nutritional status of the patient; S3: GMFS and demographic, clinical and educational characteristics of the study group population at SNS l’Arboç according to educational itineraries; S4: Schematic representation of the main periodontal disorders explored; S5: Nutritional intake of students including data from a total group and age groups; S6: Pictures of the most relevant conditions associated with poor oral health in these children. How to perform the OHI-S; S7: Summary of the protocol of hydration program; S8: Communication and fundraising campaign in the city of Mataró entitled “Food Cures”; S9: Link to a presentation in English with the description of the full intervention at L’Arboç School; S10: Educational program on OD and nutrition of children in SNS for parents, caregivers and all professionals of the school.
