# Supplementary material for: Assessment of Swallowing Disorders, Nutritional and Hydration Status, and Oral Hygiene in Students with Severe Neurological Disabilities Including Cerebral Palsy
Source: Nutrients. 2021 Jul 14;13(7):2413. doi: 10.3390/nu13072413 (PMC8308512; doi:10.3390/nu13072413)
Supplement: Supplementary file 1 [file nutrients-13-02413-s001.zip › nutrients-1258959-supplementary.pdf]

SUPPLEMENTARY MATERIAL

SM 1: A further explanation of the Rationale, Hypothesis and Aim of the Study in the context of the Program for Management of Malnutrition and Swallowing Disorders at l'Arboç School.

Arboç School is 3 km away from the Hospital de Mataró in the City of Mataró, Catalonia, Spain (45 Km north Barcelona).

Videoclip 1. L'Arboç School and the needs of their people.

Link Arboç School

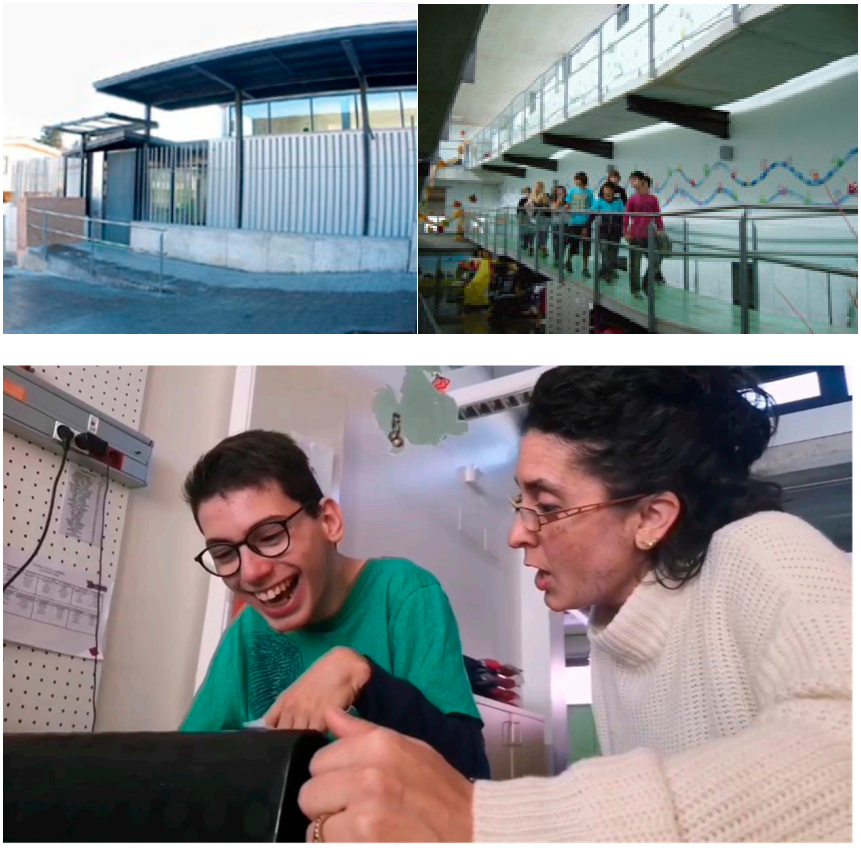

Figure S1. Images of Arboç School

The Dysphagia Unit at Mataro Hospital is one of the most active dysphagia units in Europe. The multidisciplinary team working in this Unit is composed of 20 people: a PI, several physicians (2 gastroenterologists, 2 GI Surgeons, 2 neurologists and 1 neurophysiologist), 3 SLPs, 3 dietitians, 3 research nurses, 2 nursing aids, and 2 postdoctoral researchers, and 12 of them are PhD students. The Director of this Dysphagia Unit is Prof. Pere Clavé, Director of Research and Innovation of the Hospital of Mataró and Founding President of the European Society for Swallowing Disorders.

https://orcid.org/0000-0002-0696-8560

https://scholar.google.es/citations?user=JL5NHd0AAAAJ&hl=es

Our dysphagia unit is focused on 3 main activities: a) clinical management of patients with oropharyngeal dysphagia; b) education, hosting a Masters in Swallowing Disorders with the Universitat Autonoma de Barcelona https://www.dysphagiamasters.org/en/home/, and c) Research with top level accreditation by Catalan (SGR) and Spanish authorities (Ciberehd). https://www.ciberehd.org/en

The group is formed by 7 senior researchers -5 full professors-, 3 postdoctoral researchers and 19 fellows from the Hospital de Mataró and the Universitat Autónoma de Barcelona (Department of Physiology and joined the Research Consortium Ciberehd (Spanish Ministry of Science) in 2007. https://www.ciberehd.org/

In 2019 the PI and some members of the team visited l'Arboç school and we were concerned by a) the severity of swallowing, nutrition and developmental impairments in these children; b) and the lack of tools and resources available to the teachers and professionals of this educational center that is affiliated to the Catalan Ministry of Education -not related to healthcare-, and c) the heterogeneity of the clinical management of these children as they received individualized prescriptions regarding diets and thickening agents coming from different hospitals and specialists.

We were so concerned about these 3 factors that we invited an expert on these pathologies (Katrina McLeod, an SLP from UK with long experience in SNS) and decided to undertake a program to improve the management and prognosis of these children as part of our commitment to these citizens with OD. The Hospital de Mataró bore the cost of the intervention as part of their social responsibility program.

Our plan had 3 main elements:

a) A communication and fundraising campaign in the city entitled "Food Cures'' with specific elements to increase the awareness of OD and malnutrition in these children in cooperation with Fundació Maresme (the foundation that owns and manages the school l'Arboç). You can find information on this campaign in this link in Supplementary Material 2 (sorry, it is in Spanish and Catalan but it is very clear)

https://elmenjarcura.csdm.cat/?doing_wp_cron=1621178655.7778179645538330078125

b) A clinical program with 4 elements to improve the management of these children:

1) A transversal descriptive study that will be repeated at the end of each school year (this manuscript summarizes the results of the first edition of these studies).

2) A nutritional program that includes the adaptation of texture modified foods according to a strategy we published some time ago in *Nutrients* that we call "Triple Adaptation of the Mediterranean Diet for patients with OD". The first author of this study (AC) is the same as of the present article. Costa a. Nutrients. 2019 Feb 18;11(2):425. doi: 10.3390/nu11020425

3)- A hydration program including a proactive offer of fluids at regular times, fluid thickening and the use of specially adapted cups for these children

4) An educational program on OD and nutrition of children in SNS for parents, caregivers and all professionals (teachers, assistants, SLP and nurses) of the school. You can explore in Supplementary Material (SM3) the online educational materials of this program at: https://formacio.csdm.cat:1080/login/ (User: convidat ; Password: Convidat*1)

Our hospital (Hospital de Mataró, Consorci Sanitari del Maresme), the Fundació Maresme (the owner and entity responsible for l'Arboç School), Furega (a Foundation that promotes awareness on swallowing disorders) and the City Council (Ajuntament de Mataró) have signed a MoU to guarantee the economic support of this clinical program. Other sponsors of this program are: Aigües de Mataró SA (the water supply company in the city), Nestlé Health Science SA, Lifemere, the Research Consortium, CIBERehd (through a strategic action on oropharyngeal dysphagia).

We also developed an awareness and fundraising campaign in 2019 called "Food Cures" to promote research in dysphasia and improve the health of the population

Videoclip 2. The Food Cures (El Menjar Cura) - Testimonial video, family of the Arboç School Link Food Cures

Videoclip 3. The Food Cures (El Menjar Cura) Campaign

Link Food Cures 1

Link Food Cures 2

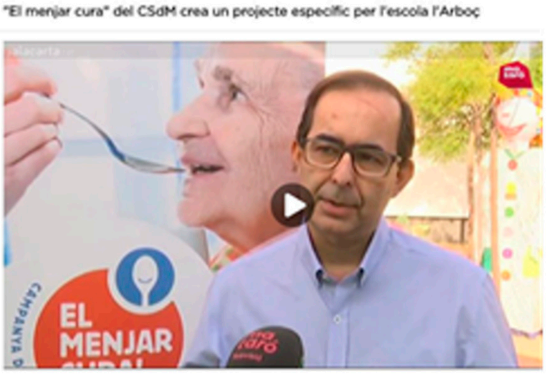

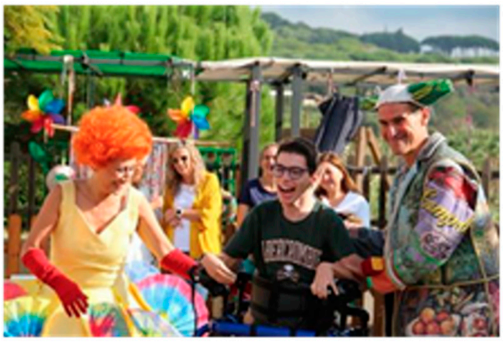

Figure S2. Images from the "Food Cures" Campaign

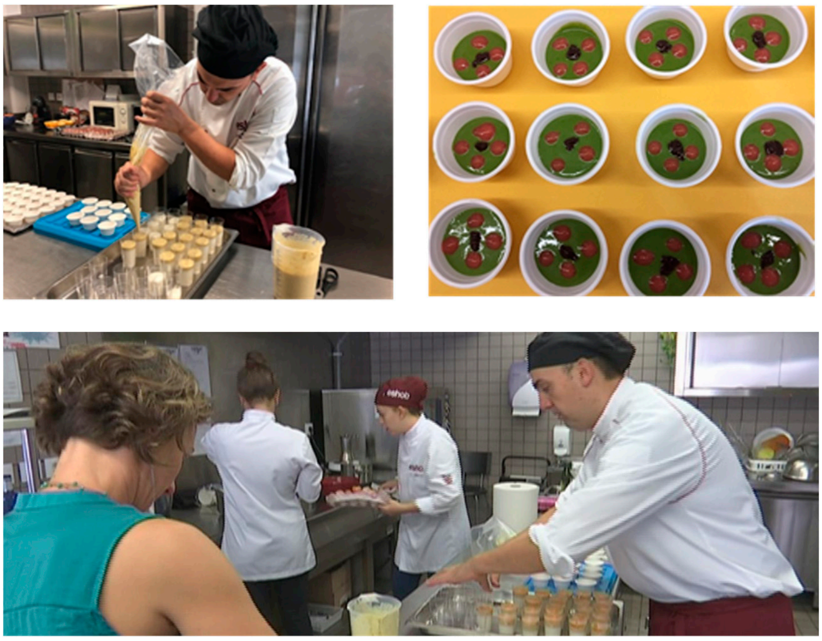

Figure S3. Pictures of the texturised food cooking workshop held at l'Arboç School (ESHOB cook preparing texturised Cuban-style rice; Texturised salad).

Videoclip 3. Videoclip gala Dinner. Food Cures

Link Gala dinner "Food Cures"

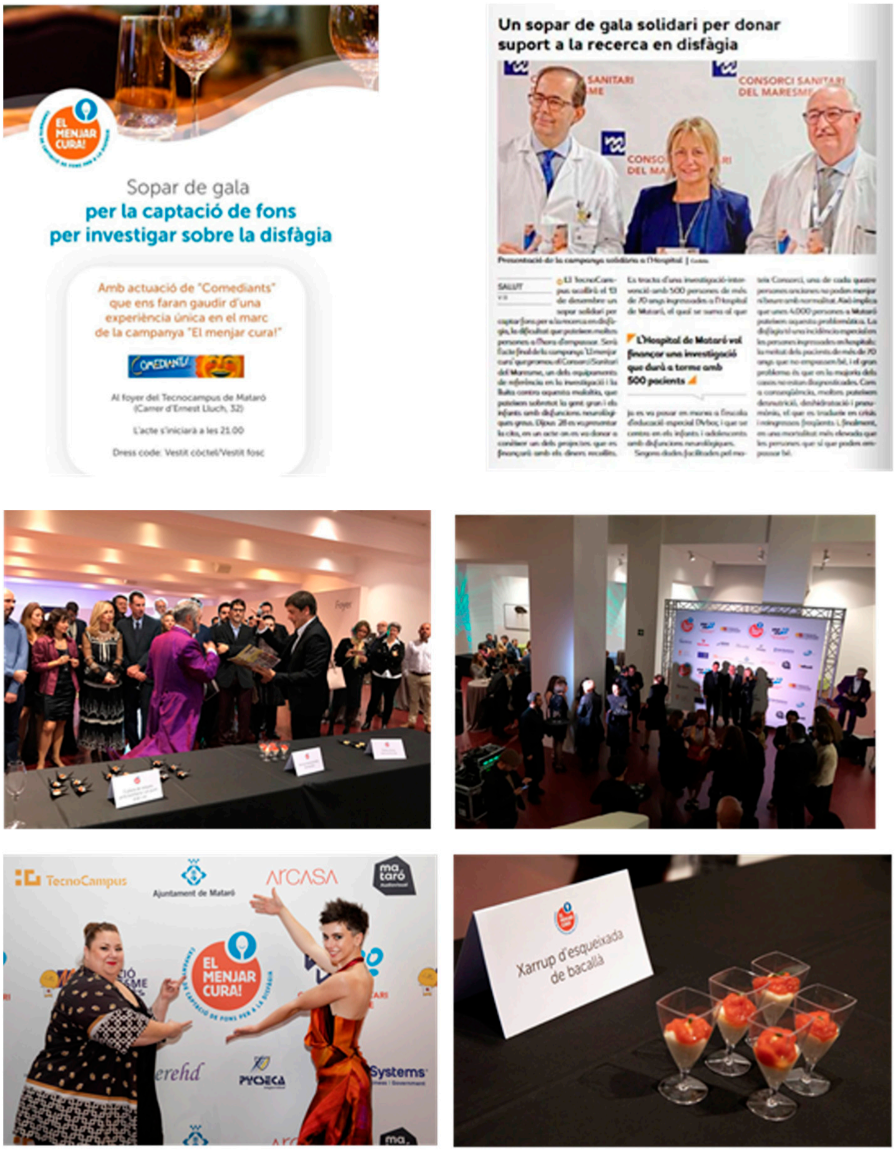

Figures S4. Gala dinner. Food Cures

Videoclip 4. Ciberehd promotes patient centered research at l'Arboç School

Link Ciberehed presentation

Web S1. Educational website for patients, relatives, caregivers and l'Arboç professionals

https://formacio.csdm.cat:1080/login/ (User: convidat ; Password: Convidat*1).

Web S2. Furega Videorecipes and tutorials.

www.furega.com (access code: Ind_2021).

The hypothesis of our study is awareness and management of OD, MN and OH in SNS in Spain is scarce and must be improved. As most of the students have severe neurological impairments, we hypothesized most of them would have dysfunctional swallowing. We believe the introduction of a program mainly based on management of OD and MN in children attending these schools and education of the same of their carers will improve their hydration and nutritional status, reduce complications associated with OD -respiratory infections, hospital readmissions-,and improve the knowledge of parents, caregivers and professionals at these schools on the management of these relevant conditions.

The aim of this study was to assess the prevalence of swallowing and feeding disorders and oral health impairments in students at l'Arboç special needs school and their relation to student's nutritional and hydration status

Our final aim is to improve the health and quality of life of all these children at special needs schools and not only the specific phenotype of CP. We believe this addition helps to clarify the overall aim of our program and we also believe this is a quite unique program in our country

The program is supported by Fundació Salut del CSdM (Link Food Cures 1), Furega (https://furega.com/disfagia/empassarbe/ ; www.furega.com), Nutricia Danone, Nestlé Healthscience (https://www.lifemere.com/shop/rosecup-with-sipper) , Aigues de Mataró, PECT Mataró-Maresme Ajuntament de Mataró and the Diputació de Barcelona

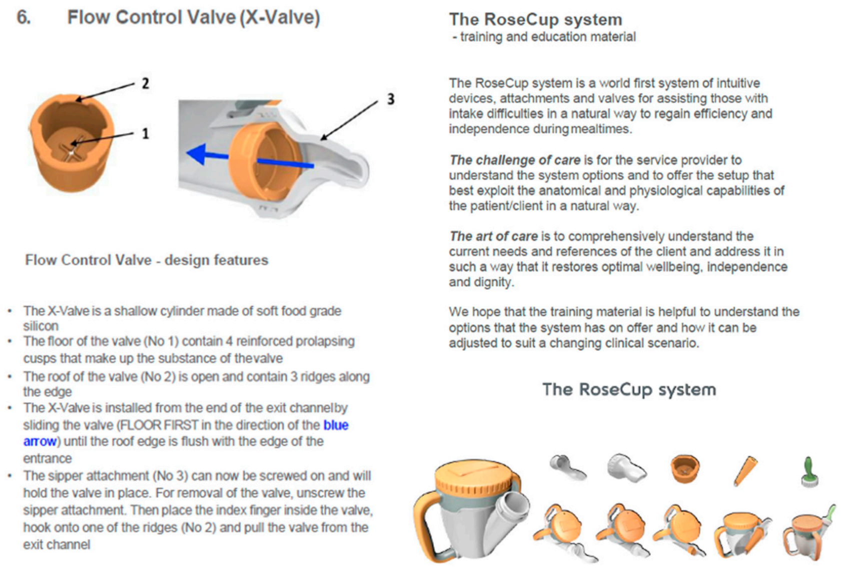

Figure S5. Images of the RoseCups:

2. **SM2.** Access to educational materials with more than 100 video recipes of the more than 250 recipes, adapted according to the degree of dysphagia and the nutritional status of the patient. These

recipes are tutorials for parents and caregivers to reproduce texture-modified dishes for the students at home. Please explore the link www.furega.com (access code: Ind_2021).

3. **SM3**. GMFS and demographic, clinical and educational characteristics of the study group population at SNS l'Arboç according to educational itineraries.

The assessment of the GMFCS and functional abilities described a severely impaired population study group:
a) 13 patients (39.4%) were classified as GMFCS level 5. They need to be transported in a wheelchair in all settings and are limited in their ability to maintain antigravity head, trunk postures and control leg and arm movements.
b) 8 (24.2%) classified as GMFCS level 4. They need methods of mobility that require physical assistance or powered mobility in most settings. They may walk for short distances at home with physical assistance or use powered mobility or a body support walker when positioned. At school, outdoors and in the community, children are transported in a manual wheelchair or use powered mobility.
c) 5 (15,2%) as GMFCS 3. They can walk using a hand-held mobility device in most indoor settings. They may climb stairs holding onto a railing with supervision or assistance. They use wheeled mobility when traveling long distances and may self-propel for shorter distances.
d) 7 (21.2%) as GMFCS 2. These students can walk in most settings and climb stairs holding onto a railing. They may experience difficulty walking long distances and balancing on uneven terrain, inclines, in crowded areas or confined spaces. Children may walk with physical assistance, a handheld mobility device or use wheeled mobility over long distances and they have only minimal ability to perform gross motor skills such as running and jumping.
e) None had GMFCS 1 as this category includes children with mild disease that can walk at home, school, outdoors and in the community. They can climb stairs without the use of a railing and perform gross motor skills such as running and jumping, but speed, balance and coordination are limited.

The school offers four work educational groups, one for each itinerary and another that combines the relational and instrumental itinerary (Group 3). The majority of students (48.5%) belong to the relational route, and only 18% of students are in the basal route. All of them belong to the highest level of disability (GMFCS=V). We observed that age or the main diagnosis were not the only criteria for classifying this group population in the school (Table 1).
Age and itinerary or route of each student are the two main variables used by the SNS to group the students for their educational needs. Based on age, they are grouped in three levels (young, 3 to 9 years; medium, 9 to 15 years, and older, 15 to 23 years). Once this first orientative grouping has been made, each student's learning route is assessed, and they are re-distributed into four groups. Basal itinerary (Group 1) is where the learning work is focused on communication, interaction, the development of each student, as well as work on their senses to bring them closer to their immediate environment. Relational itinerary (Group 2) focuses on students with difficulties to be in contact with the environment and is centred on the search for strategies that help them manage their emotions and regulate their behaviour. The instrumental curriculum itinerary (Group 4) focuses on the deployment of an adapted school curriculum. Group 3 is a mixture of children from different relational and curriculum itineraries. The itineraries are orientative and on a pedagogical level they help focus on the individual programming of each student. The same student can have work objectives of more than one itinerary, and a student can change itinerary if there are any changes in the circumstances of his/her environment. The social and cultural realities of each student were also assessed.

Table S1. Demographic, clinical and educational characteristics of the study group population at SNS l'Arboç according to educational itineraries.

	**Group 1**	**Group 2**	**Group 3**	**Group 4**	**Total**
n (students, %)	6 (18.2)	16 (48.5)	4 (12.1)	7 (21.2)	33 (100)
Age range, years	5.3 - 20.9	7.3 - 19	6.6 - 11.7	5 - 23.8	5.3- 23.8
Mean age, years	11.7 ± 6.1	13.6 ± 3.6	9.4 ± 2.6	16.3 ± 6.3	13.3 ± 4.9
GMFCS, mean	V	II-V	III-IV	III-V	II-V
Main disease, n students	CP (5) Epileptic encephalopathy (1)	CP (6) Epileptic encephalopathy (3) Hereditary diseases (4) Others (3)	CP (2) Hereditary diseases (1) Others (1)	CP (6) Other (Meningoencephalitis (1)	CP (19) Epileptic encephalopathy (4) Hereditary diseases (5), Others (5)
Education Aims	Basal itinerary	Relational itinerary	Relational and curriculum itinerary	Instrumental curriculum itinerary	SNS

GMFCS: gross motor function classification system; CP: cerebral palsy; SNS: special needs schools.

4. **SM4**. Schematic representation of the main periodontal disorders explored.

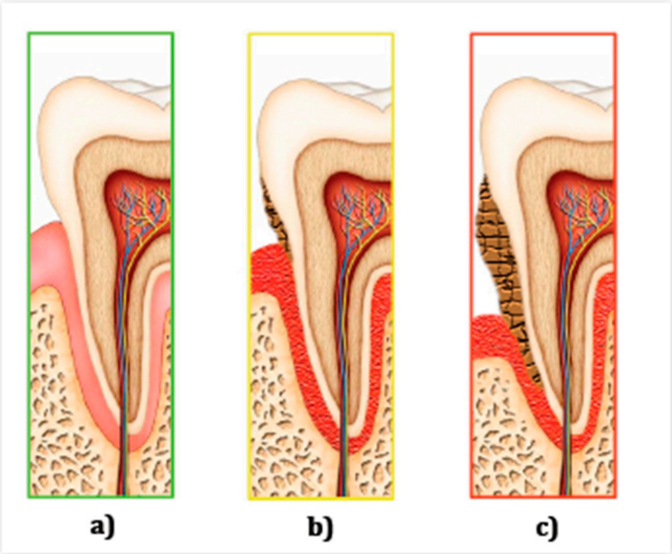

Figure S6. Evolution of periodontal disease. (**a**) Healthy periodontium with no inflammation (gingivitis) and or bone loss (periodontitis); (**b**) periodontal inflammation (gingivitis), there is still no bone loss; (**c**) Periodontitis with inflammation and bone loss. Adapted from Ortega O 2014.

- Ortega O, Parra C, Zarcero S et al. Oral health in older patients with oropharyngeal dysphagia. Age and Ageing 2014;43(1):132-7.

5. **SM5.** Nutritional intake of students including data from a total group and age groups 5-12 and 13-23 ages.

	Total Sample (SD) n=29	Intakes of students 5-12y n=15	Recommended intakes (group 1)	Intakes of students 13-23y n= 14	Recommended intakes (group 2)
Mean of days	*5 (3.7)*	*5.4 (4)*		*4.6 (3.6)*	
Energy, kcal [77]	1664.2 (629.7)	1460.2 (484.4)	1459.4± 295.4	1854.6 (703.3)	1488.1± 528.3
Proteins, g [80]	71.2 (28.7)	66.7 (34.5)	20.53±5.65*	75.4 (22.3)	31.99±12.95*
Proteins, %	17.4 (5.6)	18.1 (7.6)		16.7 (2.8)	12-15
Fats, g	64.4 (30.9)	57.3 (24.4)	48.6	71.0 (35.5)	49.6
Fats, %	34.6 (7.5)	35.2 (6.3)	30.0	34.1 (8.6)	30.0
Monounsaturated Fatty Acids, g	*20.7 (13.4)*	*16.5 (9.8)*		*24.6 (15.3)*	

Monounsaturated Fatty Acids, %		*10%*	*20%*		*20%*
Poliunsaturated Fatty Acids, g	*7.2 (5.69*	*4.9 (2.8)*		*9.4 (6.7)*	
Poliunsaturated Fatty Acid, %		*3%*	*5%*		*5%*
Saturated Fatty Acid, g	*16.8 (13.4)*	*12.1 (8.7)*		*21.2 (15.6)*	
Saturated Fatty Acid, %		*7.4%*	*7-8%*		*7-8%*
Cholesterol, mg	*198.2 (137.3)*	*173.9 (102.5)*	*<300*	*220.9 (163.6)*	*<300*
Carbohydrates, g	198.2 (85.7)	167.9 (57.6)	>130	226.5 (99.2)	>130
Carbohydrates, %	47.5 (7.5)	46.3 (6.4)	55.0	48.7 (8.5)	50-60
Sugar, g	80.6 (65.9)	54.7 (32.9)	<18	104.8 (79.9)	<18
Sugar, %	19.4	15		22.6	
Fibre, g	16.2 (8.4)	13.4 (6.9)	27.5	18.9 (9)	25-30
Water, ml [75]	1034.6 (437.6)	862.9 (436.6)	1600-2100	1195 (385.8)	2100-2500
Sodium, mg	1166.1 (729.7)	1004.9 (686.4)	1350.0	1316.6 (759.7)	1500.0
Potassium, mg	2292.2 (1207)	1901.9 (998.6)	2066.7	2656.5 (1301.1)	3500.0
Calcium, mg	882.1 (352.2)	760.1 (323.8)	1150.0	995.9 (349.3)	1150.0
Magnesium, mg	228.5 (128.2)	177.1 (97.9)	275.0	276.4 (137.4)	375.0
Phospor, g	948.4 (527.1)	753.8 (410.5)	800.0	1130 (571)	950.0
Iron, mg	11.5 (5.8)	10.2 (5.8)	10.5	12.7 (5.8)	12.5
Zinc, mg	6.7 (4)	5.0 (2.7)	12.5	8.4 (4.4)	15.0
Vitamin A, microg.	778.8 (596.2)	650.2 (699.3)	566.7	898.8 (474.2)	1000.0
Vitamin D, microg.	4.9 (8.6)	1.9 (1.7)	15.0	7.8 (11.2)	15
Vitamin E, mg	8.5 (11.0)	4.4 (2.4)	8.3	12.3 (14.3)	11.5
Vitamin B2 (Riboflavin), mg	1.3 (0.9)	1.0 (0.7)	1.2	1.6 (1)	1.8
Vitamin B1 (Thiamin), mg	1.4 (1.8)	0.8 (0.5)	0.8	2 (2.3)	1.2
Vitamin B6, mg	1.4 (0.7)	1.2 (0.6)	1.4	1.6 (0.8)	2.0
Vitamin B9 (Folic Acid), microg	203.7 (108.6)	185.7 (107.2)	250.0	220.5 (110.9)	400.0

Vitamin B3 (Niacin), mg	13.3 (6.9)	12.1 (5.7)	13.3	14.3 (7.9)	19.0
Vitamin B12, mg	2.6 (1.5)	2.2 (1.3)	1.8	3 (1.5)	2.0
Vitamin C, mg	94.9 (72.1)	77.8 (57.1)	57.5	110.7 (82.6)	60.0

Moreiras O, Carbajal A, Cabrera L, C.C. Tablas de composición de alimentos.; 18a edición.; 2016.
*FAO/WHO/UNU, J. Protein and amino acid requirements in human nutrition: report of a joint FAO/WHO/UNU expert consultation; Geneva, Switzerland, 2002.
**Holliday MA, S.W. The maintenance need for water in parenteral fluid therapy. Pediatrics 1957, 19, 823–32.

6. **SM6:** Pictures of the most relevant conditions associated with poor oral health in these children. How to perform the OHI-S.

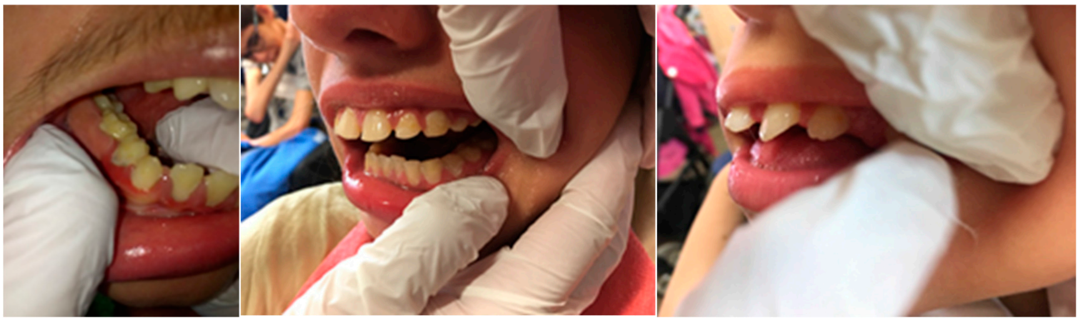

(a) (b) (c)

Figure S7. Malocclusion examples of Arboç students: (**a**) Class I, (**b**) Class II, (**c**) Class III

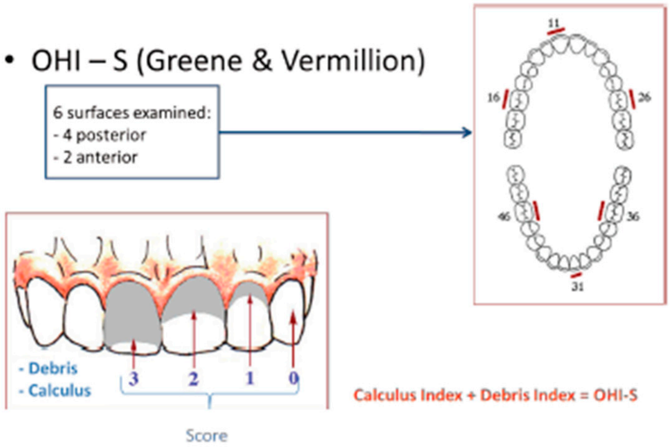

Figure S8. The oral hygiene evaluation was performed according to OHI-S developed by Greene and Vermillion. This index is a composed measurement of the accumulation of dental plaque and calculus

in 6 different teeth and is helpful to evaluate dental covering or oral biofilm of subjects assessed. We have to differentiate between debris (soft foreign matter attached to the teeth; cucin, bacteria and food) and calculus (mineralized debris made of deposits of inorganic salts). Each index is evaluated independently according to the amount of deposits found in the target tooth. Three teeth examined in the maxillary arch and 3 in the mandibular arch. We finally calculate the OHI-S by adding the results of the debris and calculus index.

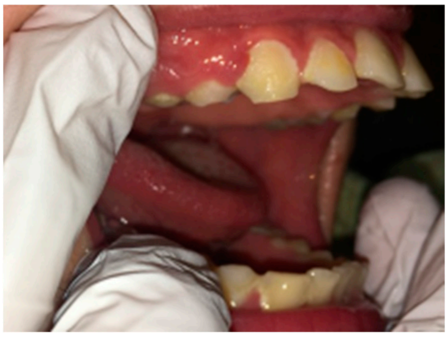

Figure S9. Periodontitis example of study sample: (a) Periodontitis Stage I Grade A, with periodontal pockets in premolars.

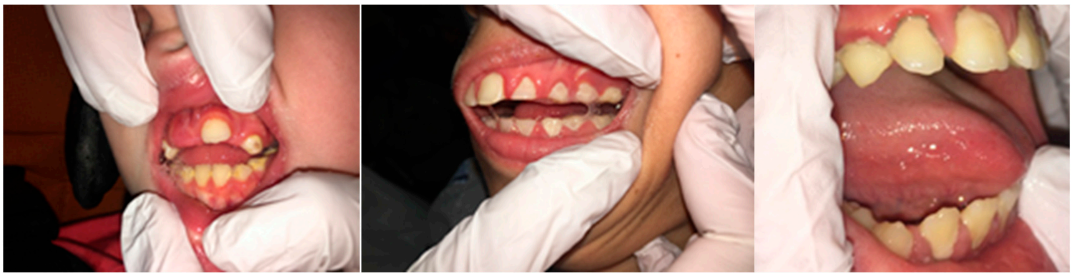

Figure S10. Gingivitis examples associated with dental plaque and calculus.

7. **SM7.** Summary of the protocol of hydration program using homogeneous (xanthan gum) fluid thickeners for all students and specific cups to guarantee the appropriate and safe fluid provision at the appropriate bolus volume and viscosity according to the V-VST (250 vs 800 mPa·s) and with specific support staff for hydration

This protocol has been approved by the Ethics Committee of the Consorci Sanitari del Maresme with code 12/20 and the study describes a prospective quasi-experimental project with the main aim to evaluate an educational dietetic intervention, specifically focused in the hydration status.

The hydration intervention consists in two phases:

1. An hydration intervention on the fluid taken

2. Educational program to school professionals, caretakers and families on dysphagia and its relationship with hydration.

For the first part, the quantity needed to give to each student will be assessed according to the age and the basal hydration status. Fluid loss (due to the labial impairment) will be offset by increasing the fluid intake calculated. In order to ensure safe swallows, a V-VST will be performed to each student and according to the results obtained, viscosity will be prescribed to 250 mPa·s or 800 mPa·s (optimal viscosities assessed by a previous study of our group (Ref. Bolívar-Prados 2019)). Specific devices such as the "RoseCup" (designed to improve swallowing in patients with OD) will be included to be used when administering the fluids.

Second part includes different theoretical sessions to introduce, describe and explain how to manage dysphagia, oral hygiene and hydration requirements.

In order to analyse the impact of the hydration intervention and educational program, the hydration status will be assessed by bioimpedance prior and after the intervention and supplemented by a hydration daily register. Knowledge acquisition of care takers, professionals and families will be assessed by questionnaires.

8. **SM8.** Communication and fundraising campaign in the city of Mataró entitled "Food Cures" with specific elements to increase the awareness of OD and malnutrition in these children with CP/NI in cooperation with Fundació Maresme (the foundation that owns and manages the school l'Arboç). You can find information on this campaign in this link (sorry is in Spanish and Catalan but very clear).
https://elmenjarcura.csdm.cat/?doing_wp_cron=1621178655.7778179645538330078125

9. **SM9**. Link to a ppt in English with the description of the full intervention at l'Arboç School. https://youtu.be/mEo6ewVdYTY

10. **SM10.** Educational program on OD and nutrition of children in SNS for parents, caregivers and all professionals (teachers, assistants, SLP and nurses) of the school. You can explore the online educational materials of this program at: https://formacio.csdm.cat:1080/login/ (User: convidat ; Password: Convidat*1).
